# Integrated B and T Cell Repertoire Analysis Reveals Organized but Heterogeneous Adaptive Immunity in Chronic Experimental Stroke

**DOI:** 10.21203/rs.3.rs-10695363/v1

**Published:** 2026-08-27

**Authors:** Sangeetha Senthil Kumar, Danielle A. Becktel, Erin D. Chapman, Susan A. Whitman, Rachel Crumpacker, Jacob C. Zbesko, Daniel Berchtold, Claudia Dames, María I. Cuartero, Alicia Garcia-Culebras, Lizi M. Hegarty, Gaia Brezzo, Jennifer E. Goertz, Kristy A. Zera, Craig J. Smith, Stuart M. Allan, Christian Meisel, Andreas Meisel, María Ángeles Moro, Barry W. McColl, Josef Anrather, Marion S. Buckwalter, Helena W. Morrison, Rick G. Schnellmann, Kristian P. Doyle

**Affiliations:** 1 Department of Immunobiology, University of Arizona, Tucson, Arizona, USA; 2 Center for Stroke Research Berlin, Neuroscience Clinical Research Center, Department of Neurology with Experimental Neurology, Charité Universitätsmedizin, Berlin, Germany; 3 Centro Nacional de Investigaciones Cardiovasculares (CNIC), Madrid, Spain; 4 Institute for Regeneration and Repair, University of Edinburgh, Edinburgh, UK; 5 UK Dementia Research Institute, Institute for Neuroscience and Cardiovascular Research, University of Edinburgh, Edinburgh, UK; 6 Feil Family Brain and Mind Research Institute, Weill Cornell Medical College, New York City, New York, USA; 7 Department of Neurology and Neurological Sciences, Stanford University School of Medicine, Stanford, California, USA; 8 Department of Biomedical Sciences, University of Minnesota Medical School, Duluth, Minnesota, USA; 9 Division of Cardiovascular Sciences, School of Medical Sciences, Faculty of Biology, Medicine and Health, University of Manchester, Manchester, UK; 10 Geoffrey Jefferson Brain Research Centre, Manchester Academic Health Science Centre, Northern Care Alliance NHS Foundation Trust, University of Manchester, Manchester, UK; 11 Manchester Centre for Clinical Neurosciences, Northern Care Alliance NHS Foundation Trust, University of Manchester, Manchester, UK; 12 Institute of Medical Immunology, Charité Universitätsmedizin, and Department of Immunology, Labor Berlin – Charité Vivantes, Berlin, Germany; 13 College of Nursing, University of Arizona, Tucson, Arizona, USA; 14 Department of Pharmacology and Toxicology, College of Pharmacy, University of Arizona, Tucson, Arizona, USA; 15 BIO5 Institute, College of Medicine, University of Arizona, Tucson, Arizona, USA; 16 Departments of Neurology, Neurosurgery, and Psychology, Arizona Center on Aging, University of Arizona, Tucson, Arizona, USA

**Keywords:** Ischemic stroke, Adaptive immunity, Neuroinflammation, B-cell receptor repertoire, T-cell receptor repertoire, Clonality

## Abstract

Ischemic stroke drives prolonged accumulation of lymphocytes within the injured brain, and we recently showed that the T-cell compartment of the chronic infarct is not a random infiltrate but a reproducibly organized oligoclonal repertoire. Whether this organization is a broader property of the post-stroke adaptive immune response, extending to B cells and coordinated across lymphocyte lineages, has remained unknown. Here we performed immunoglobulin heavy-chain (IGH) repertoire sequencing of chronic infarct tissue across different cohorts spanning age, sex, experimental stroke models, and independent study centers, and integrated these data with T-cell receptor β (TRB) repertoires from the same lesions. Chronic infarcts exhibited increased B-cell receptor clonality relative to matched splenic repertoires, a pattern reproducible across age, sex, models, and centers, yet heterogeneous among individual lesions owing to variable expansion of dominant clonotypes. Dominant B-cell clonotypes showed recurrent V(D)J gene usage across animals, and a subset of recurrent receptor frameworks contained public CDR3 amino-acid sequences despite substantial overall sequence diversity. Integrated analysis revealed that B- and T-cell repertoire concentration was positively associated within individual infarcts while remaining heterogeneous in its relative organization across lesions, and complementary immunoglobulin light-chain analyses supported the overall pattern of localized B-cell remodeling. Together, these findings show that persistent adaptive immunity after stroke is organized across multiple hierarchical levels, from clonal concentration to recurrent receptor architecture and paired B- and T-cell repertoire organization, yet remains biologically heterogeneous across individuals. This organizational framework, together with the publicly deposited repertoire dataset, provides a foundation for future studies of the antigenic drivers and functional consequences of chronic post-stroke adaptive immunity.

## 1. Introduction

Ischemic stroke initiates a prolonged inflammatory response that extends well beyond the acute ischemic insult. Within minutes of arterial occlusion, damaged neurons and glia release danger-associated molecular patterns and reactive oxygen species, activating microglia and promoting cytokine production, blood-brain barrier disruption, and secondary tissue injury [1–4]. Over subsequent days, neutrophils, monocytes, and lymphocytes infiltrate the injured brain and further shape the inflammatory milieu [5]. During the following weeks, innate and adaptive immune cells adopt dynamic, context-dependent states that can exacerbate injury or support inflammation resolution, tissue remodeling, angiogenesis, and remyelination [6, 7]. Host factors, particularly aging and infection, further modify these responses by altering leukocyte composition and function [8–10]. Stroke is therefore increasingly recognized as a chronic neuroinflammatory condition involving sustained interactions between innate and adaptive immunity.

Within this evolving neuroinflammatory response, adaptive immunity contributes to long-term tissue remodeling and neurological outcome. T cells accumulate in infarcted tissue during the acute phase and can persist for weeks to months after injury [11–14]. Their effects are context-dependent: FOXP3^+^CD4^+^CD25^+^ regulatory T cells suppress excessive inflammation and promote neuroprotection [11], whereas CD8^+^ cytotoxic T cells mediate antigen-dependent neuronal injury [15] and contribute to post-stroke white matter demyelination [16]. Clinical studies further indicate that adaptive immune responses to central nervous system (CNS) antigens develop after stroke, with stronger Th1 responses to myelin basic protein independently associated with worse functional outcomes [17]. We recently showed that T cells persisting within chronic infarcts form a reproducibly organized, infarct-associated oligoclonal repertoire conserved across age, sex, ischemic models, and independent laboratories, while paired peripheral repertoires remain broadly polyclonal [18]. These findings indicate that persistent post-stroke T-cell responses are structured rather than stochastic and raise the question of whether comparable organization extends to B cells.

Compared with T cells, the role of B lymphocytes in chronic stroke remains less well understood [19]. In addition to producing antibodies, B cells regulate immune responses through antigen presentation, cytokine production, and modulation of T-cell activity [20]. Their effects after stroke are temporally and functionally diverse. During the acute and recovery phases, IL-10-producing regulatory B cells can limit neuroinflammation and infarct expansion [21], while B-cell N-methyl-D-aspartate (NMDA) receptor signaling and mature brain-derived neurotrophic factor (BDNF) production may support neuroplasticity outside the ischemic core [22]. In contrast, chronic accumulation of B cells and plasma cells within infarcts, together with local immunoglobulin deposition, has been associated with persistent inflammation, neurodegeneration, and cognitive impairment, effects that can be mitigated by CD20^+^ B-cell depletion [23]. Clinical evidence likewise supports sustained humoral immune activation after stroke, including intrathecal immunoglobulin synthesis [24–26] and anti-neuronal autoantibodies associated with impaired memory recovery [27]. Together, these findings suggest that B-cell responses can support recovery in some contexts but contribute to chronic neurological dysfunction in others.

Despite growing evidence of chronic B-cell involvement after stroke, it remains unknown whether the repertoire-level organization observed in T cells extends to the B-cell compartment. Clonally expanded B-cell subsets and follicle-like structures have been identified within chronic infarcts, suggesting that the post-stroke brain can support localized B-cell responses [28, 29]. However, it is unclear whether these responses reflect continued lymphocyte accumulation or an organized receptor repertoire. Specifically, we do not know whether chronic infarcts exhibit recurrent B-cell repertoire features across animals or predominantly private, lesion-specific responses, whether recurrent receptor-gene usage extends to public CDR3 sequences, or how B-cell repertoire organization relates to the accompanying T-cell response. The influence of aging and biological sex on this organization also remains largely unexplored.

To address these questions, we performed immunoglobulin heavy and light-chain repertoire sequencing of chronic infarct tissue across cohorts spanning age, sex, stroke models, and independent study sites, and integrated these data with T-cell receptor repertoires obtained from the same infarct. Building on our previous identification of infarct-associated T-cell oligoclonality [18], the present study investigates whether chronic B-cell responses exhibit comparable organization and how B and T-cell repertoires relate within individual lesions. We found that chronic post-stroke adaptive immunity is reproducibly organized yet heterogeneous, with localized B-cell clonal expansion, recurrent receptor features, and variable coordination between B and T-cell repertoires. These findings define the repertoire architecture of persistent adaptive immunity after stroke and provide a framework for investigating its antigenic drivers and functional consequences.

## 2. Materials and Methods

### 2.1 Animals

**Figure 1.**
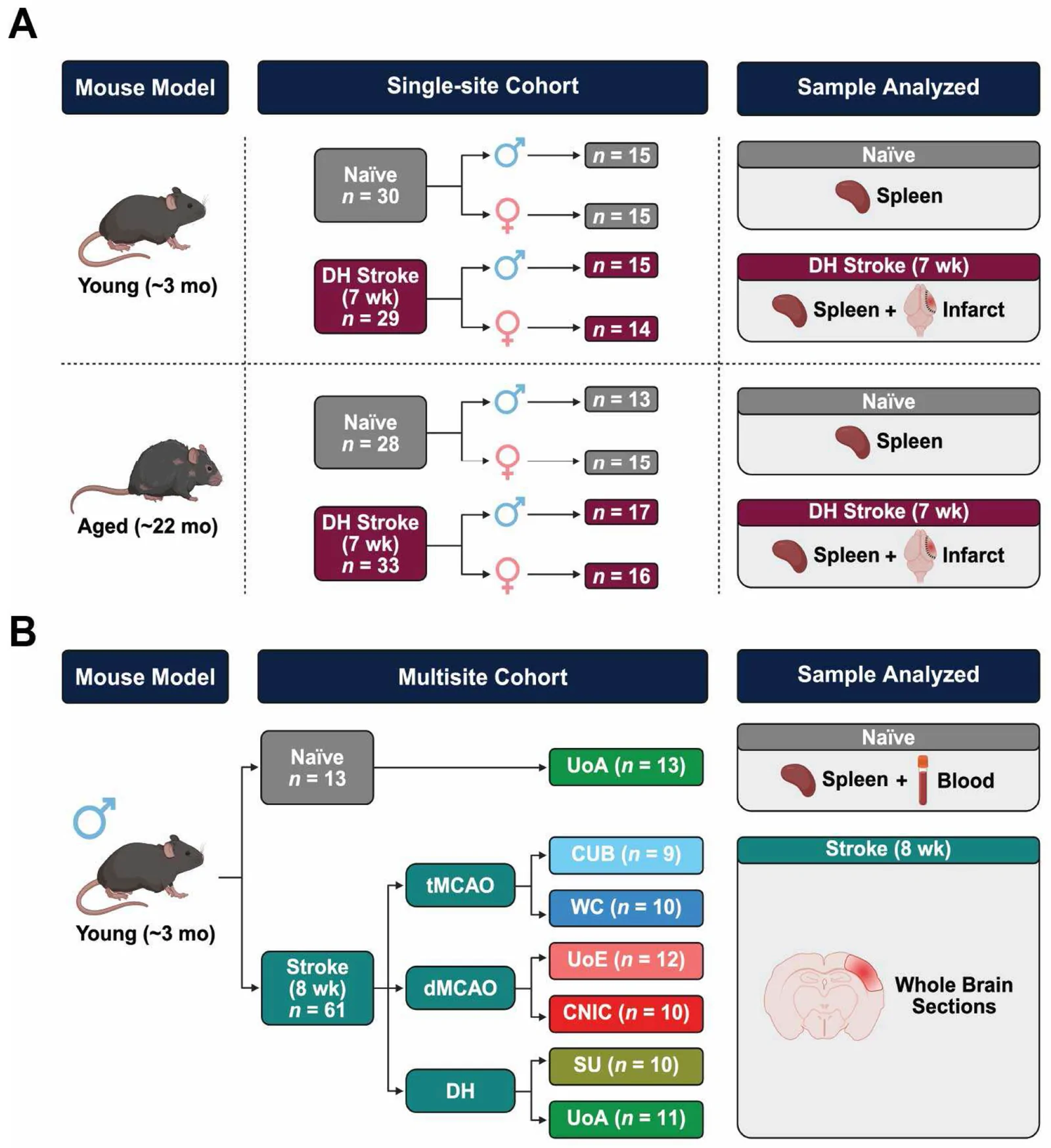
Experimental design of the single-site and multisite cohorts used for adaptive immune receptor repertoire analysis in chronic stroke. **(A)** Experimental design of the single-site cohort. Young (3-month-old) and aged (22-month-old) male and female C57BL/6 mice were randomly assigned to naïve or ischemic stroke groups. Chronic infarct tissue and matched spleens were collected 7 weeks after stroke for immunoglobulin heavy- and light-chain repertoire sequencing and histological analyses. **(B)** Experimental design of the multisite validation cohort. Chronic infarct tissue was sequenced from 61 male C57BL/6 mice across three experimental stroke models: transient middle cerebral artery occlusion (tMCAO), distal middle cerebral artery occlusion (dMCAO), and distal middle cerebral artery occlusion + hypoxia (DH). Experiments were performed independently at six research centers: Charité Universitätsmedizin Berlin (CUB), Weill Cornell Medicine (WC), University of Edinburgh (UoE), Centro Nacional de Investigaciones Cardiovasculares (CNIC), Stanford University (SU), and University of Arizona (UoA). Peripheral blood and spleen from naïve mice served as baseline controls. Multisite tissues were collected 8 weeks after stroke. Both cohorts were used to investigate the organization of adaptive immune repertoires in chronic experimental stroke.

All animal experiments were performed in accordance with institutional and national regulations and complied with the ARRIVE [30] and RIGOR [31] guidelines. C57BL/6 mice were used throughout the study (Fig. 1). The single-site cohort included young adult (3 months old) and aged (22 months old) male and female mice, whereas the multisite cohort consisted of young adult male mice only. Animals were housed under a 12-hour light/dark cycle with ad libitum access to food and water.

### 2.2 Experimental Design

The single-site cohort comprised naïve and stroke groups stratified by age and sex (120 mice total), with infarct tissue and matched spleens collected 7 weeks after stroke (Fig. 1A). The multisite cohort comprised 61 chronic infarct IGH repertoires generated using three complementary experimental stroke models: transient middle cerebral artery occlusion (tMCAO), permanent middle cerebral artery occlusion (dMCAO), and distal hypoxic stroke (DH) [32, 33]. These studies were independently performed at six laboratories: Charité-Universitätsmedizin Berlin (CUB, Germany), Weill Cornell Medicine (WC, USA), University of Edinburgh (UoE, UK), Centro Nacional de Investigaciones Cardiovasculares (CNIC, Spain), Stanford University (SU, USA), and University of Arizona (UoA, USA). Peripheral blood and splenic samples from naïve control animals (*n* = 13) were included for baseline comparison, yielding a total of 74 repertoires for multisite IGH analysis (Fig. 1B). Multisite samples were collected 8 weeks after stroke. Animals were randomly assigned to experimental groups before surgery. Samples submitted for adaptive immune receptor sequencing were coded so that sequencing personnel were blinded to experimental group, stroke model, and study site.

### 2.3 Ischemic Stroke Models

#### Single-site cohort.

Chronic infarcts in the single-site cohort were generated using the DH stroke model, performed as detailed in the accompanying T-cell repertoire study [18]. Briefly, mice were anesthetized with isoflurane and maintained at 37°C, and buprenorphine (0.1 mg/kg, subcutaneous) was administered preoperatively for analgesia. The right middle cerebral artery (MCA) was exposed through a temporoparietal burr hole and permanently occluded by cauterization. Immediately after occlusion, mice were placed in a hypoxia chamber for 45 min; young adult mice were exposed to 9% oxygen and aged mice to 11% oxygen to account for age-related differences in ischemic tolerance. Extended-release buprenorphine (Ethiqa XR, 3.25 mg/kg, subcutaneous) was administered 24 h after surgery. At the experimental endpoint (7 weeks after stroke), mice were anesthetized with isoflurane and transcardially perfused with 0.9% saline, and infarct tissue and matched spleens were collected for downstream analysis.

#### Multisite cohort.

Chronic infarcts in the multisite cohort were generated using three complementary ischemic stroke models, each independently performed at six laboratories, with each model reproduced at two sites, as described in the accompanying T-cell repertoire study [18]. tMCAO was performed using the intraluminal filament method at CUB (60 min occlusion) and WC (25 min occlusion). dMCAO was performed at UoE and CNIC. DH stroke was performed at SU (60 min hypoxia) and UoA (45 min hypoxia). Detailed information on the site-specific surgical procedures has been described previously [34, 35]. All procedures were approved by the institutional animal care and use committee or equivalent regulatory body at each participating site and complied with relevant national and institutional regulations. Infarct tissue was collected 8 weeks after stroke, and peripheral blood and spleen from naïve control animals served as baseline references.

### 2.4 Immunohistochemistry

B220 immunohistochemistry was performed on free-floating mouse brain sections to visualize B-cell distribution within chronic infarcts. Sections were washed in phosphate-buffered saline (PBS, pH 7.4), and endogenous peroxidase activity was quenched by incubation in hydrogen peroxide prepared in 0.3% Triton X-100/PBS for 20 min at room temperature. After blocking with 5% normal goat serum containing 0.3% Triton X-100/PBS, tissues were incubated overnight at room temperature with biotin rat anti-mouse B220/CD45R (Clone RA3-6B2) (BD Biosciences, Cat. No. 553085) diluted in 3% goat serum/PBS. Following washing, immunoreactivity was amplified using an avidinbiotin complex (ABC) kit (Vector Labs, Cat. No. PK-7100). Signal was visualized with a 3,3′-diaminobenzidine (DAB) substrate kit (Vector Labs, Cat. No. SK-4100), after which sections were mounted, dehydrated through graded alcohols, and cover slipped using standard procedures.

### 2.5 BCR Repertoire Sequencing and Analysis

For the single-site cohort, genomic DNA was isolated from chronic infarct and matched splenic tissues and submitted to CD Genomics for bulk B-cell receptor (BCR) immunosequencing. For the multisite cohort, genomic DNA was isolated from fixed chronic infarct tissue sections, and immunoglobulin heavy-chain (IGH) repertoire sequencing was performed by Adaptive Biotechnologies. DNA extractions were performed as previously published [18]. Rearranged IGH and immunoglobulin kappa (IGK) and lambda (IGL) light-chain loci were amplified by multiplex PCR targeting V, D (IGH only), and J gene segments spanning the complementarity-determining region 3 (CDR3). Sequencing libraries were prepared using Illumina-compatible adapters and sequenced as paired-end 150-bp reads.

Raw sequencing reads from the single-site cohort were processed using MiXCR (v3.0.6) for V(D)J assignment and clonotype reconstruction against the IMGT *Mus musculus* reference database. Default MiXCR parameters, including built-in error correction, were used throughout, and samples with V(D)J assignment rates >70% were retained for downstream analyses. Processed repertoire files were analyzed using custom Python scripts. Productive IGH, IGK, and IGL clonotypes were extracted, whereas rearrangements containing premature stop codons (*) or incomplete codons (_) were excluded. Clone fractions were normalized so that the summed abundance of productive clonotypes equaled one for each sample. Repertoire diversity was quantified using Simpson diversity $D=\sum_{i=1}^{n}p_{i}^{2}$ where *p_i_* represents the relative abundance of the *i*th productive clonotype, and clonality was defined as √D. For the multisite cohort, IGH repertoire datasets from Adaptive Biotechnologies were analyzed using the same downstream computational workflow to enable direct comparison with the newly generated single-site BCR repertoires.

To characterize dominant clonal architecture, productive IGH clonotypes were ranked according to clone fraction within each repertoire, and the ten most abundant productive clonotypes were selected for downstream analyses. These Top-10 productive clonotypes were used for stacked bar visualizations of dominant clonal composition. Analyses of recurrent IGH V(D)J frameworks and public CDR3 clonotypes were performed using these ranked productive clonotypes from the single-site dataset, whereas the multisite cohort was used to assess the reproducibility of IGH clonality across independent stroke models and laboratories.

### 2.6 Clone-level treemap visualization.

Treemap visualizations were generated to illustrate the clonal architecture of individual immunoglobulin heavy-chain (IGH) repertoires using a standardized Python-based squarified treemap pipeline previously developed for T-cell receptor (TCR) repertoire analysis [18]. The previously published TCR repertoires from the corresponding experimental cohorts were reanalyzed, enabling direct qualitative comparison of BCR and TCR repertoire architecture [18]. Briefly, productive clonotypes, defined as individual productive B-cell receptor rearrangements identified from the immunosequencing dataset and characterized by a productive CDR3 amino acid sequence, associated V(D)J gene assignment, and clone fraction, were extracted. Nonproductive rearrangements were excluded. Within each repertoire, clonotypes sharing identical productive CDR3 amino acid sequences were collapsed into unique amino acid clonotypes by summing their corresponding clone fractions. The collapsed repertoire was subsequently renormalized to generate relative clone fractions prior to treemap construction. Each rectangle represents one unique productive CDR3 amino acid clonotype after collapsing identical sequences, with rectangle area proportional to its normalized relative abundance within the repertoire. A consistent rank-based color scheme, fixed dimensions, proportional scaling, and uniform styling were applied across all samples to facilitate direct qualitative comparison of repertoire organization. Treemaps were generated for every individual mouse across the entire cohort to visualize the clonal architecture of naïve spleen, stroke spleen, and chronic infarct repertoires. These whole-cohort visualizations enabled qualitative comparison of repertoire organization, clonal dominance, and tissue-specific repertoire architecture across biological groups and were subsequently interpreted alongside the corresponding paired T-cell repertoires to assess similarities and differences in adaptive immune organization.

### 2.7 Analysis of Recurrent IGH and TRB V(D)J Frameworks

Recurrent V(D)J framework analyses were performed using the single-site cohort. Productive IGH and previously generated productive T-cell receptor β (TRB) repertoires from chronic infarct tissue were analyzed independently within each biological cohort (young male, young female, aged male, and aged female). For each animal, productive clonotypes were ranked by clone fraction without amino acid collapsing, and the ten most abundant productive clonotypes, ranked R1-R10, were selected for analysis.

For every top-ranked clonotype, V, D, and J gene assignments were extracted and concatenated to generate a receptor-gene framework (IGHV|IGHD|IGHJ or TRBV|TRBD|TRBJ). Within each biological cohort, recurrent frameworks were defined as identical V(D)J combinations detected among the top ten clonotypes of at least two mice, whereas frameworks identified in only one animal were classified as private.

Cohort-specific heatmaps were generated with individual mice represented as rows and clone-rank positions (R1–R10) represented as columns. Recurrent V(D)J frameworks were assigned cohort-specific identifiers (V1, V2, V3, etc.), with each identifier denoting a complete recurrent V(D)J gene combination. Frameworks detected in only one mouse were classified as private and retained as individual V(D)J combinations. Horizontal bar plots summarized the number of mice in which each recurrent framework was detected. IGH and TRB repertoires were analyzed independently using the same analytical workflow to enable direct comparison of recurrent V(D)J framework usage between B-cell and T-cell repertoires.

### 2.8 Analysis of Public Clonotypes and CDR3 Diversity

Analyses were performed using the single-site cohort. Recurrent IGH and TRB V(D)J frameworks identified from the Top-10 productive clonotype analysis were ranked according to the number of mice in which they occurred. The three most prevalent recurrent frameworks from each receptor repertoire were selected for downstream public clonotype and CDR3 diversity analyses and designated V1–V3 for visualization. For each selected framework, all productive clonotypes carrying the corresponding V(D)J combination were extracted from infarct repertoires across all biological cohorts. The number of mice carrying each framework and the number of unique amino-acid CDR3 sequences associated with that framework were determined to quantify sequence diversity. For each selected framework, all productive clonotypes carrying the corresponding V(D)J combination were extracted from infarct repertoires across all biological cohorts. The number of mice carrying each framework and the number of unique amino-acid CDR3 sequences associated with that framework were determined to quantify sequence diversity.

Public clonotypes were defined as identical productive amino-acid CDR3 sequences detected in at least two mice within the same recurrent V(D)J framework. For each recurrent framework, identical amino-acid CDR3 sequences were grouped across animals to determine the number and proportion of public clonotypes relative to the total number of unique amino-acid CDR3 sequences. The most broadly shared public clonotype was identified according to the number of mice in which it was detected, and nucleotide diversity underlying the shared amino-acid sequence was assessed by determining the number of unique nucleotide CDR3 sequences encoding that clonotype.

To evaluate tissue distribution, the occurrence of each selected recurrent V(D)J framework among the ten most abundant productive clonotypes was quantified independently in naïve spleen, stroke spleen, and chronic infarct repertoires. For each tissue compartment, the number of mice containing the framework within their dominant repertoire was determined.

Results were summarized using bubble plots illustrating the relationship between V(D)J recurrence, amino-acid CDR3 diversity, and public clonotypes, bar graphs depicting the proportion of public clonotypes within each recurrent framework, bar graphs showing the most broadly shared public clonotypes together with their underlying nucleotide diversity, and bar graphs comparing the distribution of recurrent V(D)J frameworks across tissue compartments.

### 2.9 Statistical Analysis

Statistical analyses were performed using GraphPad Prism v11. Data were first tested for normal distribution with the Shapiro-Wilk (W) test. For data demonstrating normal distribution, parametric tests were applied, specifically: (1) a two-tailed, unpaired *t* test for comparisons between two groups or (2) an ordinary one-way ANOVA followed by Dunnett’s multiple comparisons test for comparisons between multiple groups. In cases where data did not exhibit normal distribution, nonparametric tests were utilized: (1) a Mann-Whitney *U* test for comparisons between two groups or (2) a Kruskal-Wallis test followed by Dunn’s multiple comparisons test for comparisons between multiple groups. Data are presented as mean ± SD. Spearman correlation analysis was conducted to assess the association between paired IGH and TRB clonality measurements, and results were represented graphically using curves indicating 95% confidence intervals. Statistical significance was defined as *P < 0.05, **P < 0.01, ***P < 0.001, ****P < 0.0001.

## 3. Results

### 3.1 IGH repertoires undergo tissue-associated clonal remodeling within the chronic infarct.

**Figure 2.**
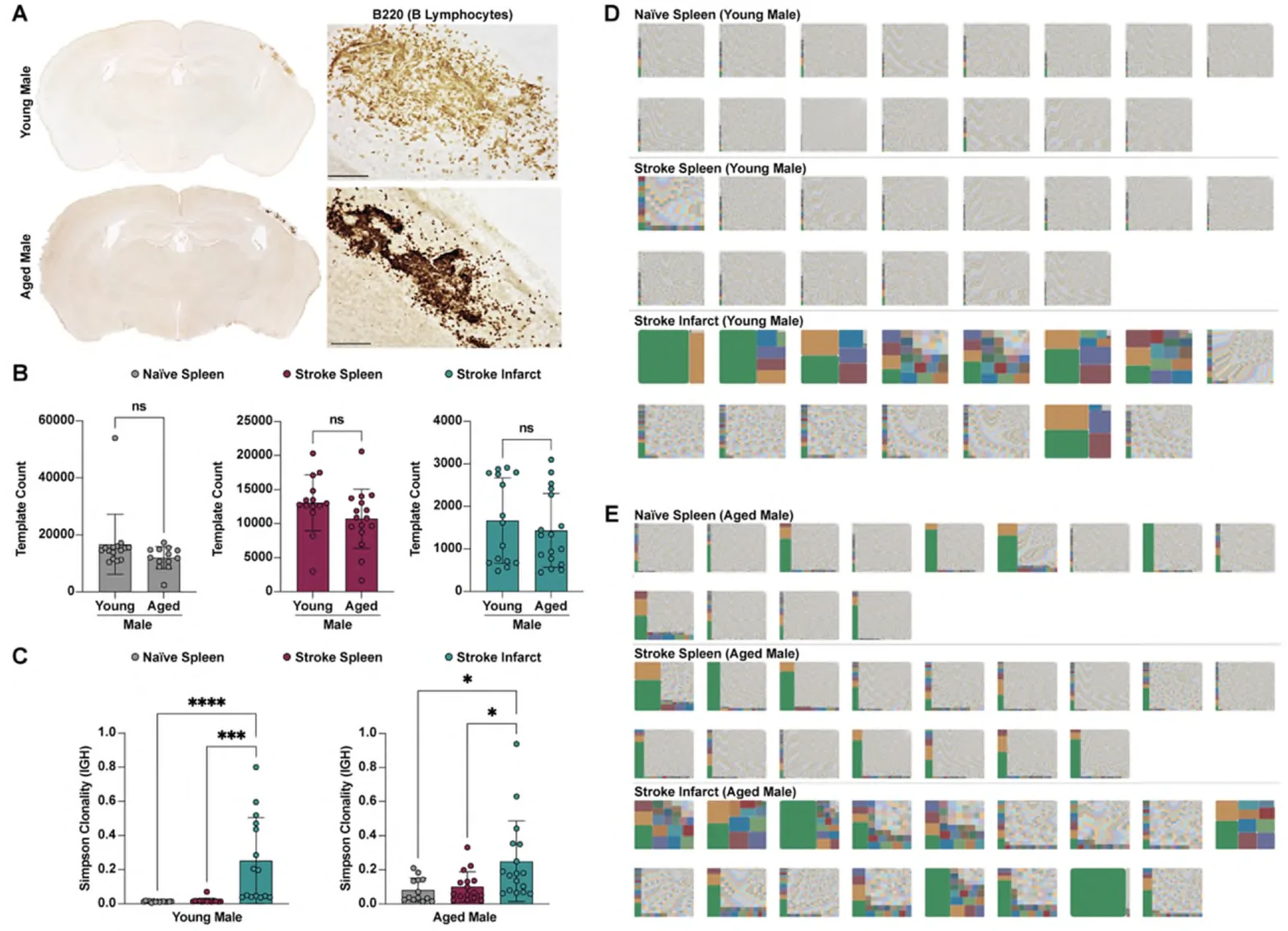
Chronic stroke is associated with selective IGH repertoire remodeling within the infarct. **(A)** Representative whole-brain sections and higher-magnification images showing B-cell accumulation within infarcts at 7 weeks post-stroke in young and aged mice. Scale bars, 125 μm. **(B)** Productive IGH template counts in naïve spleen (gray) and in paired spleen (maroon) and infarct (teal) samples from young and aged male mice subjected to DH stroke. Stroke spleen and infarct tissues were collected from the same animals. **(C)** Simpson clonality of productive IGH repertoires in naïve spleen, stroke spleen, and infarct samples from young and aged cohorts. **(D, E)** Treemaps of productive IGH repertoires from individual **(D)** young male and **(E)** aged male mice. Each treemap is from a different mouse and each rectangle represents a unique productive IGH clonotype, with rectangle area proportional to its relative abundance within the repertoire. Data are presented as mean ± SD. ns, not significant. *P < 0.05; ***P < 0.001; ****P < 0.0001.

At 7 weeks after stroke, B cells were detected within the infarct region in both young and aged male mice (Fig. 2A). To determine whether B-cell accumulation in the chronic lesion was associated with altered B-cell receptor (BCR) repertoire organization, productive immunoglobulin heavy-chain (IGH) repertoires were analyzed in paired spleen and infarct tissues from male mice subjected to DH stroke. Spleens from naïve male mice were included as controls.

Productive IGH template recovery, reflecting repertoire sampling depth, was comparable between young and aged mice within each tissue compartment, with no significant age-related differences in naïve spleen, stroke spleen, or infarct repertoires (Fig. 2B). Despite comparable repertoire sampling across age groups, infarct IGH repertoires exhibited significantly higher Simpson clonality than splenic repertoires in both young and aged mice (Fig. 2C). Because spleen and infarct samples were obtained from the same mice, this compartmental divergence indicates that increased IGH clonality is preferentially associated with the chronic infarct rather than with uniform contraction of the systemic B-cell repertoire.

Treemap visualization further illustrated the distinct repertoire architecture of the chronic infarct (Fig. 2D, E). Naïve and stroke spleen repertoires were broadly distributed across numerous clonotypes, with limited dominance by individual clones. In contrast, multiple infarct repertoires contained expanded clonotypes that accounted for substantial fractions of the productive repertoire. This pattern was observed in both young (Fig. 2D) and aged (Fig. 2E) mice, although the extent of clonal dominance varied among individual animals. Together, these findings indicate that B-cell accumulation within the chronic infarct is associated with localized remodeling of the IGH repertoire, characterized by increased clonality and selective enrichment of dominant clonotypes, while the paired splenic repertoire remains comparatively diverse. The presence of this compartment-associated pattern in both age groups suggests that chronic infarct-associated B-cell repertoire remodeling is not restricted to young animals.

### 3.2 Female mice show heterogeneous IGH repertoire architecture in chronic stroke.

**Figure 3.**
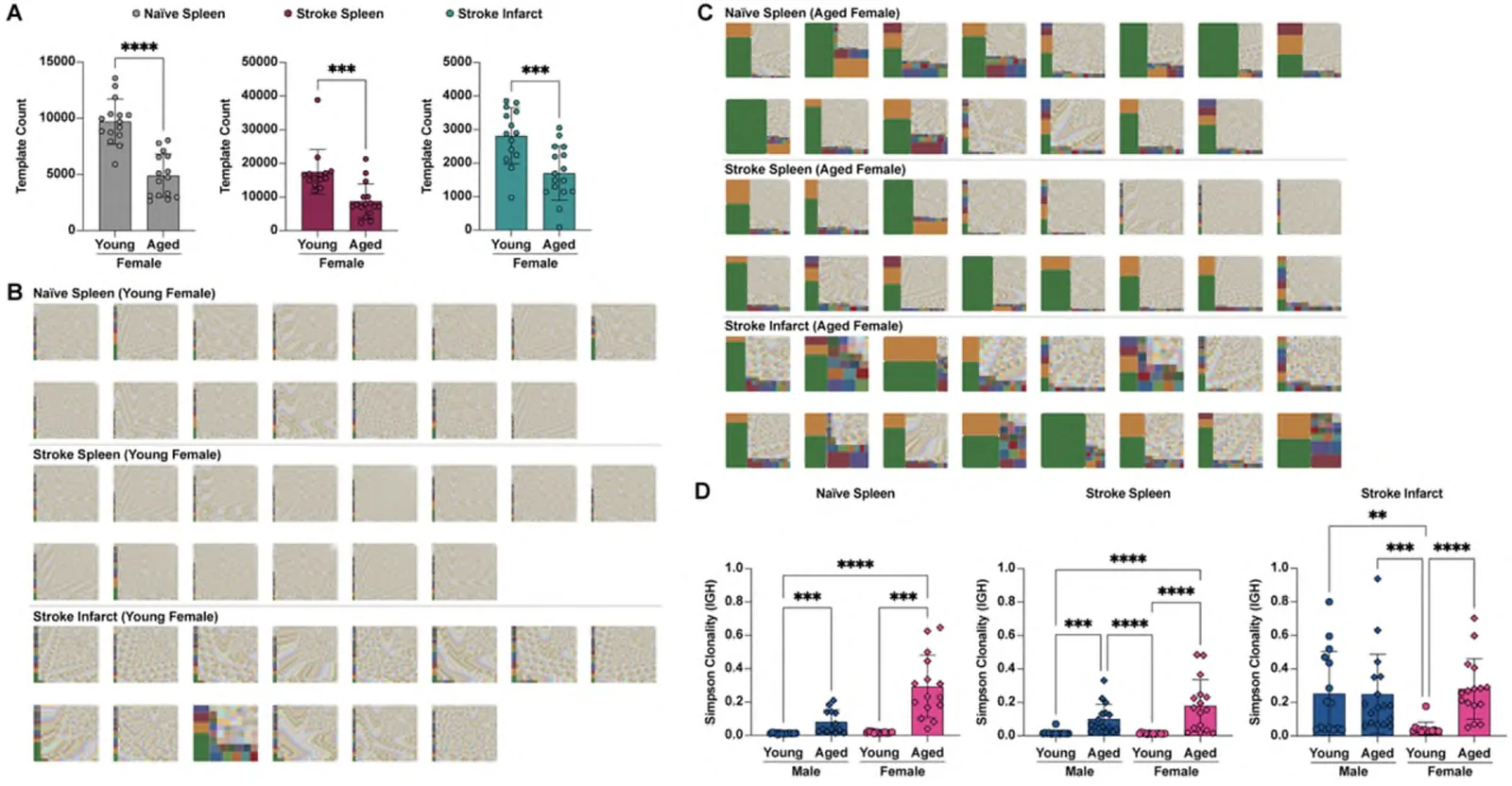
Female IGH repertoire architecture varies with age and tissue compartment. **(A)** Productive IGH template counts in naïve spleen (gray) and in stroke spleen (maroon) and infarct (teal) samples collected at 7 weeks post-stroke from young and aged female mice. **(B, C)** Treemap visualization of productive IGH repertoires from individual **(B)** young female and **(C)** aged female mice. Repertoires are shown separately for naïve spleen, stroke spleen, and infarct. Each rectangle represents a unique productive IGH clonotype, and rectangle area is proportional to its relative abundance within the repertoire. Each treemap represents one mouse. **(D)** Summary of Simpson clonality in naïve spleen, stroke spleen, and infarct repertoires across young male, aged male, young female, and aged female cohorts. Stroke spleen and infarct samples were collected from the same stroke animals. Data are presented as mean ± SD. *P < 0.05; **P < 0.01; ***P < 0.001; ****P < 0.0001.

We next examined productive IGH repertoires in young and aged female mice at 7 weeks post-stroke. Productive IGH template recovery differed significantly between young and aged females within naïve spleen, stroke spleen, and infarct compartments, with aged females showing lower template recovery across tissues (Fig. 3A).

Treemap visualization revealed substantial heterogeneity in female IGH repertoire architecture (Fig. 3B, C). In young female mice (Fig. 3B), naïve spleen and stroke spleen repertoires were largely distributed across many clonotypes. Most young female infarct repertoires also remained relatively distributed, with only one infarct sample showing marked clonal dominance. Thus, unlike the stronger infarct-associated expansion seen in male cohorts, robust IGH clonal expansion was not a uniform feature of young female infarcts.

In aged female mice (Fig. 3C), repertoire architecture was more variable across all compartments. Notably, some naïve spleen repertoires already contained dominant clonotypes, indicating that increased clonal dominance was not restricted to the infarct compartment in aged females. Stroke spleen and infarct repertoires also showed variable patterns, with some samples displaying expanded clonotypes and others remaining more distributed.

Stratification by age and sex revealed distinct patterns of IGH Simpson clonality across tissue compartments (Fig. 3D). In both naïve and post-stroke spleen, aged mice exhibited higher clonality than young mice, with the highest values observed in aged females. Within chronic infarcts, young females showed lower clonality than young males, aged males, and aged females, whereas the remaining three groups displayed broader distributions and higher mean clonality. Notably, elevated clonality in aged females was also apparent in both splenic compartments, indicating that this effect was not restricted to the infarct. Together, these data indicate that IGH repertoire concentration varies by age and sex in a compartment-specific manner, with comparatively limited clonal concentration in infarcts from young female mice.

Because the primary repertoire analyses focused on IGH, we next analyzed whether tissue-associated differences in clonality were also evident at the immunoglobulin light-chain loci. In young males, stroke infarcts showed significantly higher IGH and IGK clonality than both naïve and post-stroke spleen, whereas differences in IGL were not statistically significant. Aged males exhibited increased infarct clonality across all three loci, although the increase in IGK was significant relative to naïve spleen but not post-stroke spleen. In females, tissue-specific patterns were less consistent: young females showed increased IGH clonality in infarcts and significant differences among IGK compartments, whereas aged females showed no significant tissue differences in IGH or IGK and only higher infarct IGL clonality relative to post-stroke spleen. Together, these light-chain analyses support infarct-associated repertoire concentration most consistently in male mice, while also revealing locus-, age-, sex-, and tissue-dependent variation (Supplementary Figs. 1 and 2).

Visualization of productive top 10 clone-fraction distributions defined by distinct colors for each clone from clone 1 to clone 10 showed that IGH and IGK repertoires exhibited broadly similar patterns across biological groups, with substantial variation among individual mice in the proportion of the entire productive repertoire (Supplementary Fig. 3A,B). In contrast, IGL repertoires displayed a distinct clone-fraction distribution in which the top ten productive clonotypes consistently occupied a larger proportion of the repertoire, leaving a comparatively smaller fraction represented by all remaining productive clonotypes (Supplementary Fig. 3C). These analyses indicate that the distribution of productive clonotypes differs among immunoglobulin loci, with IGH and IGK exhibiting similar repertoire profiles that are distinct from those observed for IGL.

### 3.3 Infarct-associated IGH repertoire remodeling is robust in a multicenter, multi-model stroke cohort.

**Figure 4.**
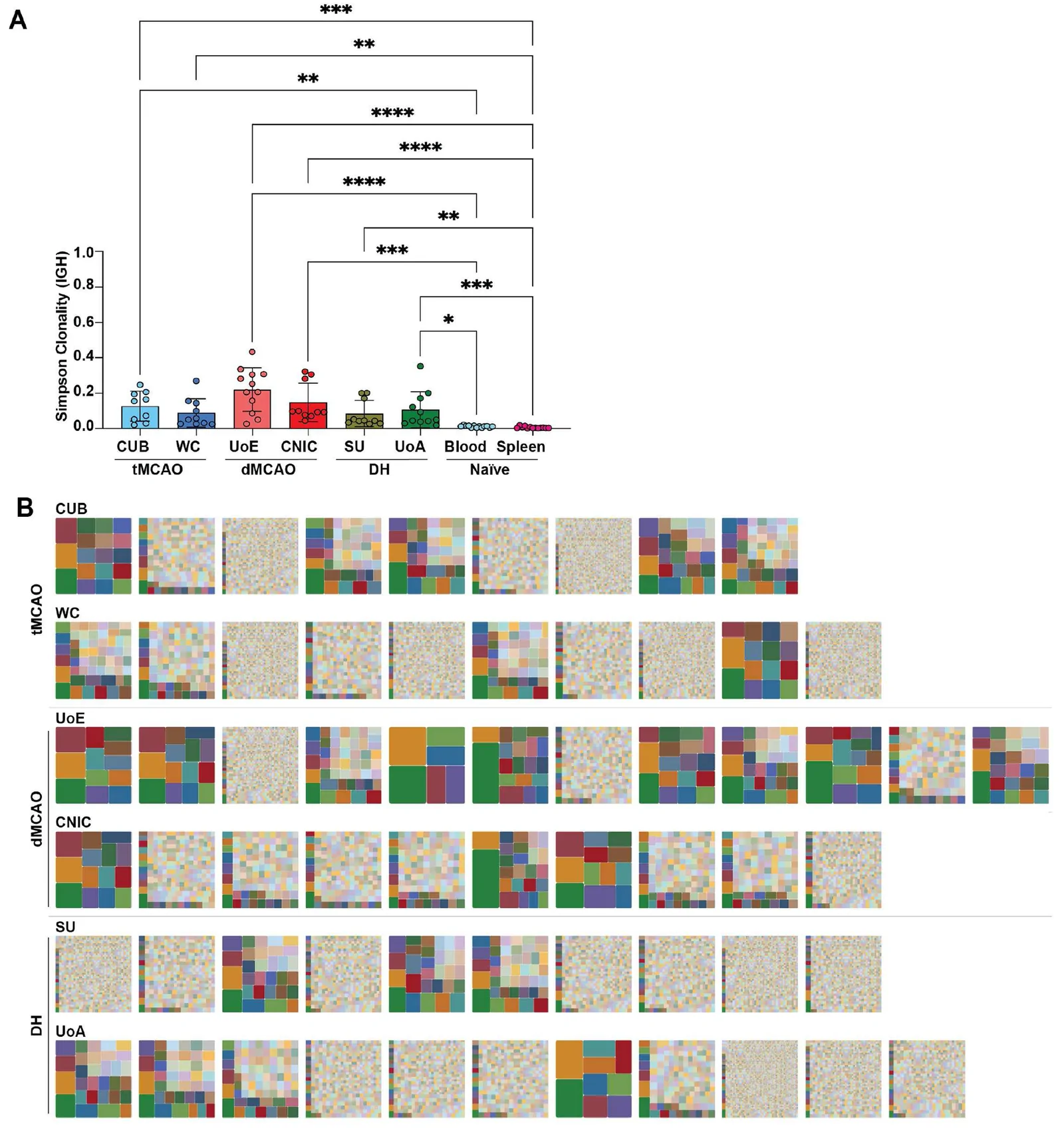
Multicenter analysis validates infarct IGH repertoire remodeling across experimental stroke models. **(A)** Simpson clonality of productive IGH repertoires from infarct tissue collected across independent experimental stroke cohorts and from naïve spleen and blood controls. Stroke models included tMCAO, dMCAO, and DH stroke. (**B)** Treemap visualization of productive IGH repertoires from individual infarct samples, organized by experimental stroke model and contributing center. Each rectangle represents a unique productive IGH clonotype, and rectangle area is proportional to its relative abundance within the repertoire. Each treemap represents one mouse. Data are presented as mean ± SD. *P < 0.05; **P < 0.01; ***P < 0.001; ****P < 0.0001. CUB, Charité Universitätsmedizin Berlin (Germany); WC, Weill Cornell Medicine (USA); UoE, University of Edinburgh (UK); CNIC, Centro Nacional de Investigaciones Cardiovasculares (Spain); SU, Stanford University (USA); UoA, University of Arizona (USA).

To determine whether infarct-associated increases in IGH clonality were reproducible across experimental settings, we analyzed productive IGH repertoires from chronic infarcts generated at multiple research centers using complementary experimental stroke models. Infarct repertoires from CUB (tMCAO), CNIC (dMCAO), and UoA (DH) exhibited significantly higher Simpson clonality than both naïve blood and spleen repertoires. In contrast, infarct repertoires from WC (tMCAO), UoE (dMCAO), and SU (DH) showed significantly higher clonality than naïve spleen repertoires but did not differ significantly from naïve blood repertoires (Fig. 4A). Collectively, these findings demonstrate that increased IGH clonal concentration is reproducibly observed across independent laboratories and complementary experimental stroke models, although the magnitude of clonality varies among sites. Together, these data indicate that increased IGH repertoire clonality is a robust and reproducible feature of chronic infarcts across independent laboratories and complementary experimental stroke paradigms, although the degree of repertoire focusing varies between sites.

Treemap visualization revealed substantial inter-animal heterogeneity both within and across experimental centers (Fig. 4B). Some infarct repertoires contained one or several dominant clonotypes that accounted for substantial fractions of the productive repertoire, whereas others retained more distributed clonal architectures. Importantly, repertoires with prominent clonal dominance were observed among samples originating from multiple centers and stroke models, indicating that this repertoire architecture was not confined to a single experimental setting.

Together, these findings extend the single-center observations to a geographically and experimentally diverse cohort and support increased IGH clonality as a robust feature of the chronic post-stroke infarct. At the same time, the marked inter-animal variation in clonal architecture indicates that the magnitude of B-cell repertoire remodeling is highly heterogeneous across individual animals.

### 3.4 Integrated BCR and TCR repertoires exhibit associated but heterogeneous clonal remodeling

**Figure 5.**
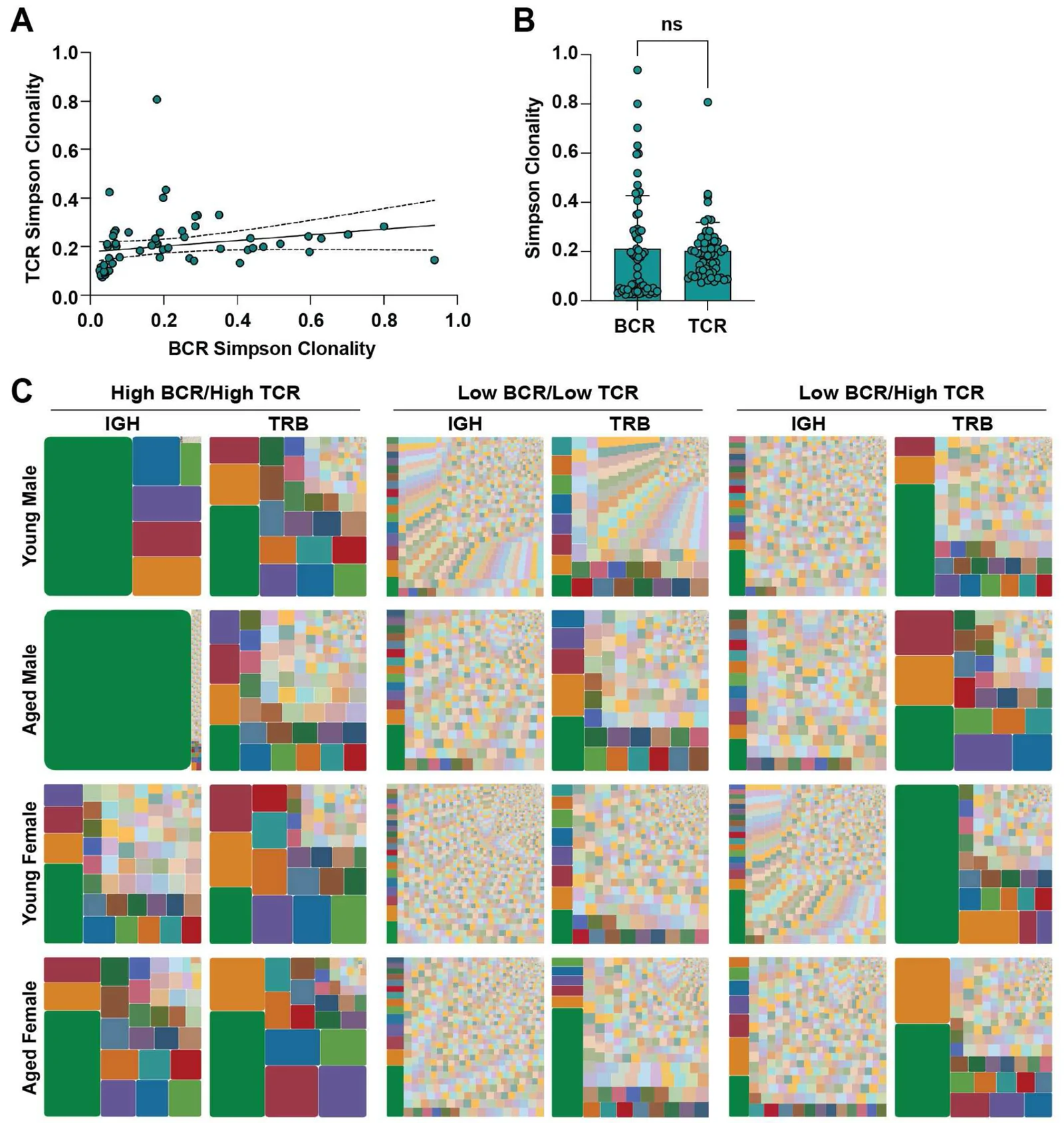
Paired BCR and TCR repertoires reveal associated but heterogeneous clonal remodeling. **(A)** Correlation between BCR and TCR Simpson clonality across matched infarct repertoires comprising young and aged male and female mice. Each point represents one animal with paired productive IGH and TRB repertoire data from the same infarct. Association was assessed by two-tailed Spearman correlation (*r* = 0.59, 95% CI 0.39-0.74, ****P < 0.0001). A fitted solid regression line with dashed 95% confidence intervals is shown for visualization. **(B)** Comparison of BCR and TCR Simpson clonality across the complete matched infarct cohort comprising young and aged male and female mice. Each point represents one mouse. Data are presented as mean ± SD. ns, not significant. **(C)** Representative same-mouse IGH and TRB repertoire treemaps selected to illustrate high BCR/high TCR, low BCR/low TCR, and low BCR/high TCR clonal architectures across young male, aged male, young female, and aged female cohorts. Each paired IGH and TRB treemap within a row was generated from the same infarct. Each rectangle represents a unique productive clonotype, with rectangle area proportional to its relative abundance within the repertoire. Complete IGH and TRB treemaps for individual mice across all age and sex cohorts are provided in Supplementary Fig. 4–7.

To determine how infarct-associated B-cell repertoire remodeling relates to our previously reported T-cell repertoire remodeling within the same lesion [18], we compared productive IGH and TRB repertoires from matched infarct samples. Across paired repertoires, IGH and TRB Simpson clonality values were positively correlated (Spearman *r* = 0.59, 95% CI 0.39-0.74, *P* < 0.0001), indicating that infarcts with more clonally concentrated B-cell repertoires also tended to contain more clonally concentrated T-cell repertoires (Fig. 5A). This association supports coordinated lesion-level variation in the extent of B- and T-cell repertoire focusing, while not establishing directionality, shared antigen specificity, or causal interaction between the two lymphocyte compartments. Alongside this positive correlation, the overall distributions of IGH and TRB Simpson clonality values did not differ significantly across matched infarct samples (Fig. 5B). Thus, at the cohort level, B- and T-cell repertoires exhibited broadly comparable degrees of clonal concentration.

However, B-cell repertoire clonality was more variable than T-cell repertoire clonality across matched infarcts. Representative paired treemaps illustrate this heterogeneity (Fig. 5C). Some infarcts exhibited pronounced clonal enrichment in both the IGH and TRB repertoires (high BCR/high TCR), whereas others displayed relatively distributed repertoires in both compartments (low BCR/low TCR). A third pattern was also evident, in which marked TRB clonal dominance was accompanied by a comparatively distributed IGH repertoire (low BCR/high TCR). Together, these findings indicate that matched infarcts do not conform to a single BCR-TCR repertoire architecture but instead exhibit distinct patterns of adaptive immune clonal remodeling.

Together, these findings identify a nuanced association between B- and T-cell repertoire remodeling within the chronic post-stroke lesion. The positive correlation between paired IGH and TRB clonality indicates that the degree of adaptive immune repertoire focusing covaries across chronic infarcts. However, the presence of discordant repertoire architectures in individual lesions indicates that this relationship is not uniform and that B- and T-cell clonal remodeling can vary independently. Together, these findings suggest that chronic infarcts differ not only in the overall magnitude of adaptive immune repertoire modelling but also in the relative contribution of B- and T-cell expansion. Complete IGH and TRB treemaps for individual mice across all age and sex cohorts are provided in Supplementary Fig. 4–7, extending the representative paired examples in Fig. 5C and illustrating the full spectrum of lesion-level BCR-TCR repertoire architectures.

### 3.5 Dominant infarct IGH clonotypes exhibit recurrent V(D)J gene usage within and across cohorts.

**Figure 6.**
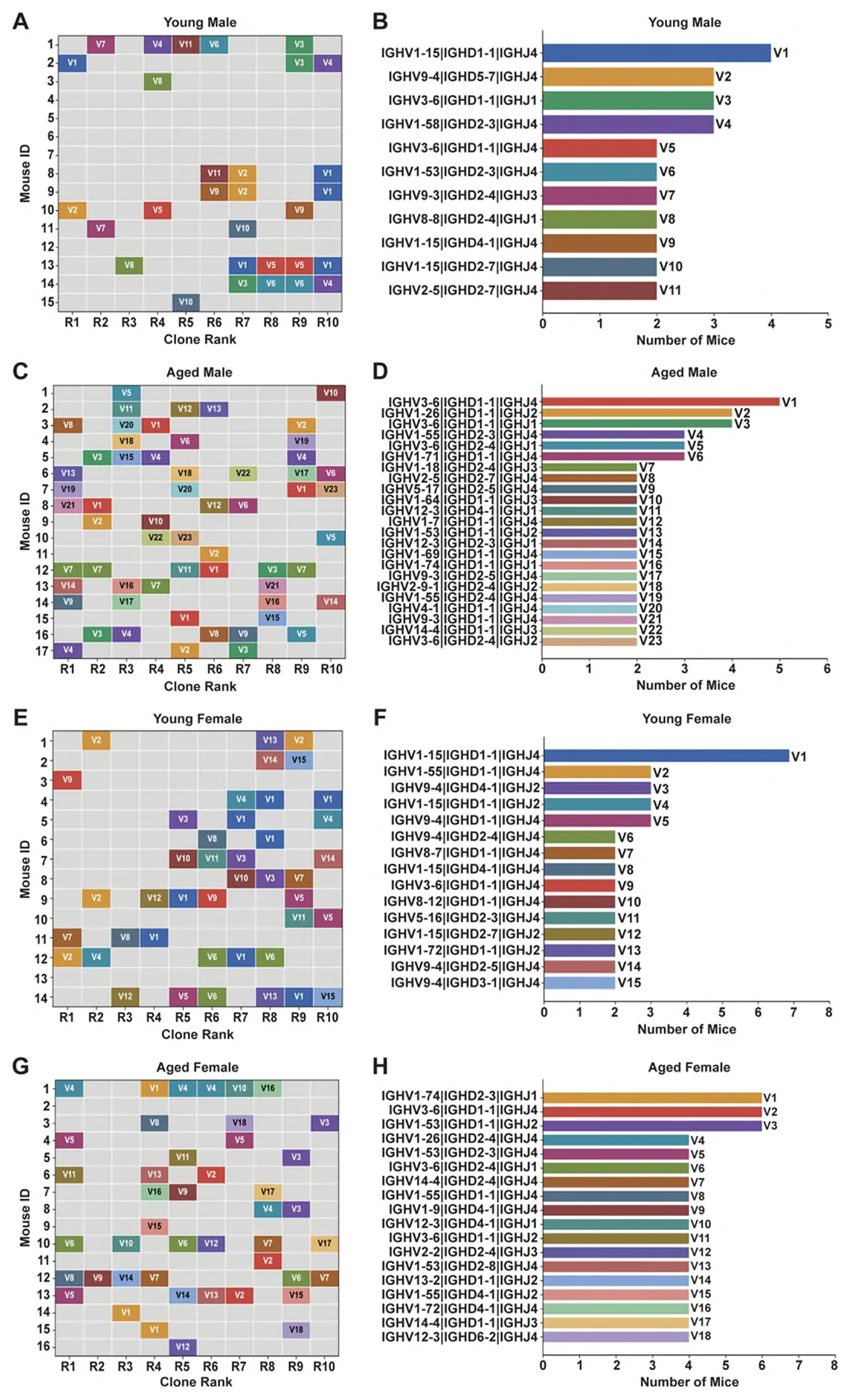
Dominant infarct IGH clonotypes exhibit recurrent V(D)J gene usage within and across cohorts. V(D)J gene combinations among the ten most abundant productive IGH clonotypes from individual infarct repertoires in the **(A, B)** young male, **(C, D)** aged male, **(E, F)** young female, and **(G, H)** aged female cohorts. Heatmaps **(A, C, E, G)** show recurrent V(D)J combinations within the ten highest-ranked IGH clonotypes of each mouse, with rows representing individual mice and columns indicating clone rank (R1-R10). Colored cells denote V(D)J combinations detected in two or more mice within the corresponding cohort, whereas gray cells denote combinations not recurrent across mice. V1-V10 within each colored cell denote cohort-specific visualization identifiers assigned to recurrent complete V(D)J gene combinations and do not represent individual V-gene segments. Bar graphs **(B, D, F, H)** show the number of mice in which each recurrent V(D)J combination was detected among the ten most abundant clonotypes. V(D)J combinations are ordered according to the number of mice in which they were observed.

To determine whether dominant B-cell clonotypes exhibited recurrent immunoglobulin gene rearrangement patterns after ischemic stroke, we compared V(D)J gene combinations among the 10 most abundant productive IGH clonotypes from each infarct repertoire across mice within each age- and sex-defined cohort. Recurrent combinations were defined as identical V(D)J gene combinations represented among the top 10 clonotypes of at least two mice within a cohort (Fig. 6).

Ranked clone maps revealed substantial diversity in V(D)J gene usage among dominant IGH clonotypes, together with recurrent use of selected combinations across animals in all four cohorts (Fig. 6A, C, E, G). Recurrent combinations occurred at different positions within the top 10 clone hierarchy and were not restricted to the most abundant clonotype. Corresponding recurrence analyses showed that some V(D)J combinations were shared by only a small subset of mice, whereas others were represented more broadly within individual cohorts (Fig. 6B, D, F, H).

Comparison across cohorts further showed that recurrent V(D)J usage was not exclusively cohort restricted. Six V(D)J combinations were represented among the top 10 infarct clonotypes of at least one mouse in each of the four cohorts. However, representation across all four cohorts did not necessarily indicate within-cohort recurrence. Notably, IGHV3-6|IGHD1-1|IGHJ4 was the only V(D)J combination recurrent in at least two mice within each cohort, identifying a consistent pattern of dominant IGH gene usage across age and sex groups. Other combinations exhibited broader or more restricted distributions across cohorts and individual animals.

Together, these findings indicate that dominant IGH clonotypes within chronic infarcts exhibit recurrent V(D)J gene usage against a background of substantial inter-animal repertoire diversity. The presence of selected combinations across biological cohorts indicates that dominant infarct repertoires are not composed exclusively of cohort-restricted rearrangement patterns. However, recurrence at the level of V(D)J gene usage does not establish convergent CDR3 sequences or shared antigen specificity, which were examined separately in subsequent analyses.

Source data underlying the ranked clone maps and recurrence analyses, including V(D)J combination identity, mouse and specimen identifiers, clone rank, clone fraction, and clone count, are provided in **Supplementary Table 1**.

### 3.6 Dominant infarct TRB clonotypes exhibit recurrent V(D)J gene usage across age and sex cohorts.

**Figure 7.**
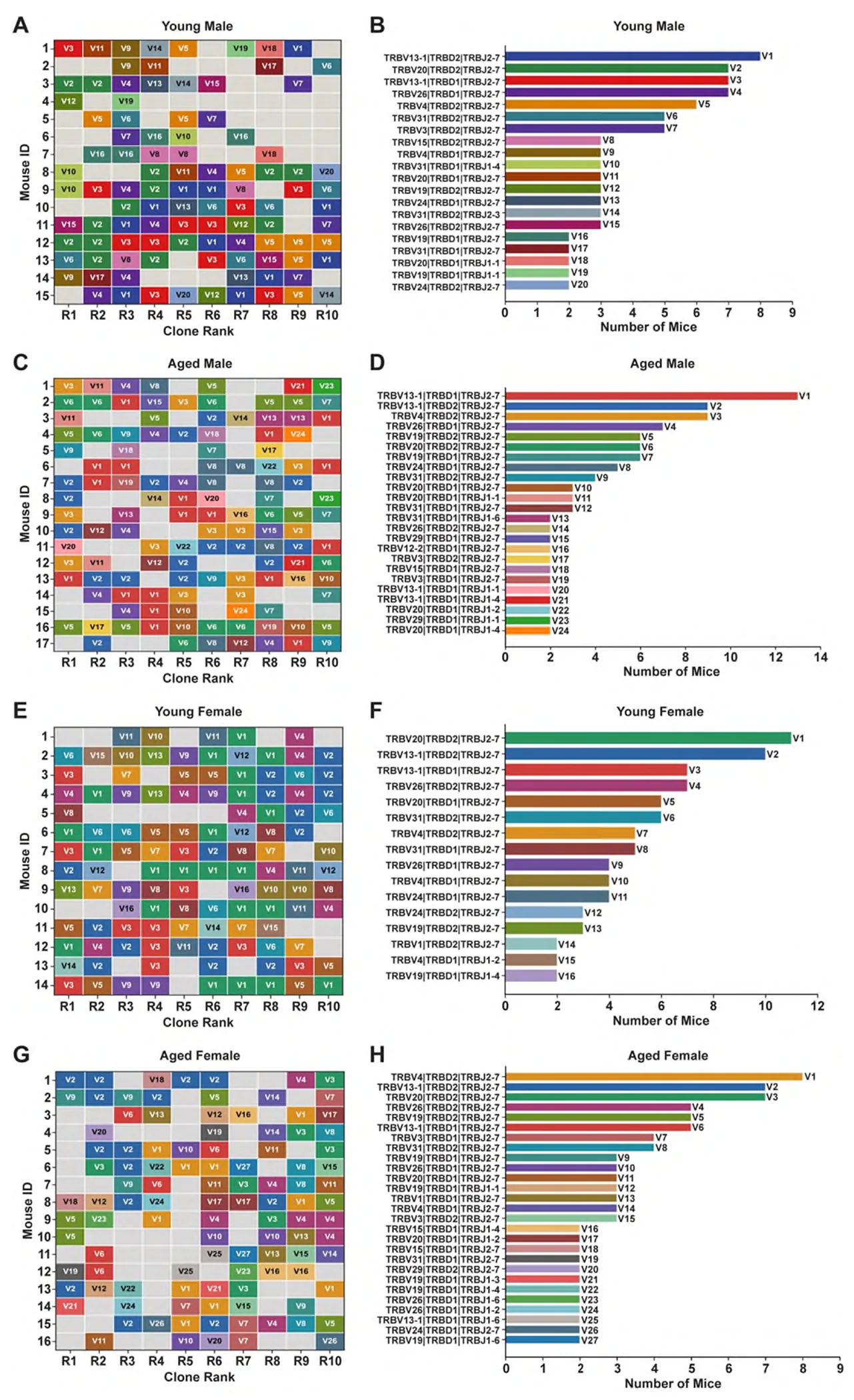
Dominant infarct TRB clonotypes exhibit recurrent V(D)J gene usage across age and sex cohorts. V(D)J gene combinations among the ten most abundant productive TRB clonotypes from individual infarct repertoires in the **(A, B)** young male, **(C, D)** aged male, **(E, F)** young female, and **(G, H)** aged female cohorts. Heatmaps **(A, C, E, G)** show recurrent V(D)J combinations within the ten highest-ranked TRB clonotypes of each mouse, with rows representing individual mice and columns indicating clone rank (R1-R10). Colored cells denote V(D)J combinations detected in two or more mice within the corresponding cohort, whereas gray cells denote combinations not recurrent across mice. V1–V10 within each colored cell denote cohort-specific visualization identifiers assigned to recurrent complete V(D)J gene combinations and do not represent individual V-gene segments. Bar graphs **(B, D, F, H)** show the number of mice in which each recurrent V(D)J combination was detected among the ten most abundant clonotypes. V(D)J combinations are ordered according to the number of mice in which they were observed.

To determine whether recurrent receptor gene usage was also evident in the T-cell compartment, we compared V(D)J gene combinations among the 10 most abundant productive TRB clonotypes from each infarct repertoire across mice within each age- and sex-defined cohort (Fig. 7). Recurrent combinations were defined as identical V(D)J gene combinations represented among the top 10 clonotypes of at least two mice within a cohort.

Ranked clone maps revealed recurrent use of selected TRB V(D)J combinations across mice in all four cohorts, together with substantial diversity in the gene usage and rank of the dominant clonotypes (Fig. 7A, C, E, G). Corresponding recurrence analyses showed that some V(D)J combinations were shared across substantial proportions of mice within individual cohorts, whereas others were recurrent in smaller subsets of animals (Fig. 7B, D, F, H).

Cross-cohort comparison identified 11 V(D)J combinations that were recurrent in at least two mice within each of the four cohorts, revealing a broadly shared component of dominant TRB gene usage across young and aged male and female mice.

Together with the IGH analysis, these findings indicate that recurrent V(D)J gene usage among dominant clonotypes is evident in both B- and T-cell repertoires within chronic infarcts. However, the extent of cross-cohort recurrence differed between the two receptor compartments. TRB repertoires contained a larger set of V(D)J combinations that met the recurrence criterion in all four cohorts, whereas IGH repertoires contained a more limited shared set against a background of cohort-variable recurrence. Thus, both adaptive immune receptor compartments exhibit recurrent patterns of dominant V(D)J gene usage but differ in the extent to which these patterns are shared across age and sex cohorts. As with IGH, recurrence at the level of TRB V(D)J gene usage does not establish convergent CDR3 sequences or shared antigen specificity.

Source data underlying the ranked clone maps and recurrence analyses, including V(D)J combination identity, cohort, mouse count, top 10 clone count, clone fraction, clone count, rank position, and mouse identifiers, are provided in **Supplementary Table 2**.

### 3.7 Recurrent IGH and TRB V(D)J frameworks contain public CDR3 sequences with distinct compartmental distributions.

**Figure 8.**
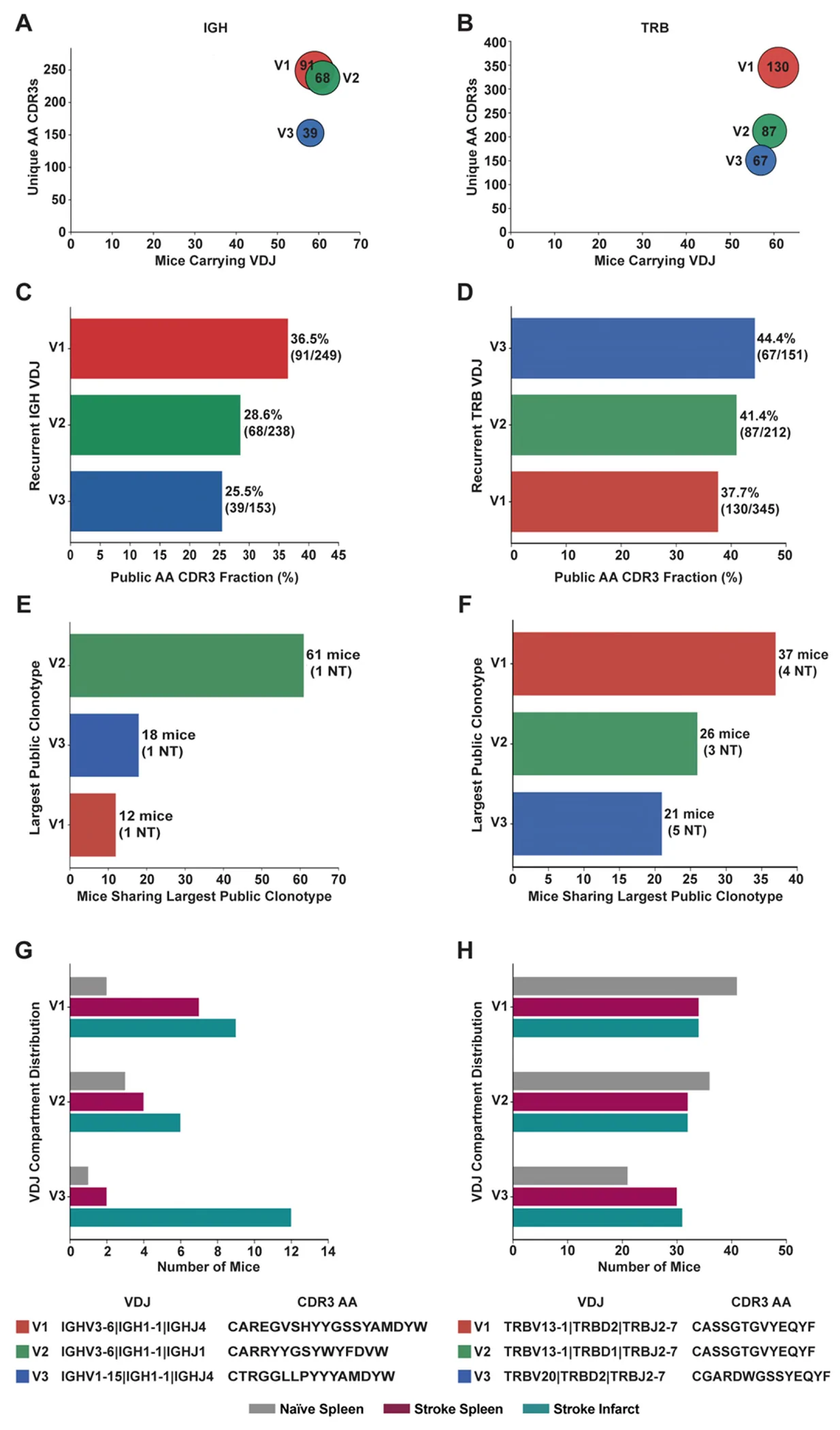
Recurrent IGH and TRB V(D)J frameworks contain public CDR3 sequences with distinct compartmental distributions. The three most recurrent V(D)J combinations from the IGH and TRB repertoires were selected according to the number of mice carrying each combination and examined for CDR3 amino-acid sequence diversity, public clonotype sharing, and tissue distribution. **(A, B)** Relationship between the number of mice carrying each recurrent V(D)J combination and the number of unique CDR3 amino-acid sequences associated with that combination for IGH **(A)** and TRB **(B)**. Bubble labels indicate V1-V3, corresponding to the top 3 recurring V(D)J combinations; numbers within bubbles and bubble size indicate the number of public CDR3 amino-acid sequences identified within each V(D)J framework. **(C, D)** Fraction of unique CDR3 amino-acid sequences that were public within each selected **(C)** IGH and **(D)** TRB V(D)J framework. Values are shown as percentages, with the number of public CDR3 amino-acid sequences over the total number of unique CDR3 amino-acid sequences in parentheses. **(E, F)** Number of mice sharing the largest public amino-acid CDR3 clonotype within each **(E)** IGH and **(F)** TRB V(D)J framework. **(G, H)** Number of mice in which the selected public clonotype from each **(G)** IGH and **(H)** TRB framework was detected in naïve spleen, stroke spleen, and infarct repertoires. V1-V3 identifiers correspond to the V(D)J combinations and CDR3 amino-acid sequences listed below the figure. Public clonotypes were defined as identical CDR3 amino-acid sequences detected in more than one mouse. NT indicates the number of distinct CDR3 nucleotide sequences encoding the indicated public CDR3 amino-acid sequence. AA, amino acid; NT, nucleotide. V1–V3 denote the three most prevalent recurrent V(D)J frameworks identified from the Top-10 productive clonotype analysis, ranked according to the number of mice in which each framework was detected.

Having identified recurrent V(D)J gene usage among dominant IGH and TRB clonotypes in chronic infarcts, we next asked whether this recurrence extended to the sharing of identical CDR3 amino-acid sequences across animals. For each receptor compartment, the three most recurrent V(D)J combinations were selected based on the number of mice in which each combination was detected and examined for CDR3 amino-acid sequence diversity and public clonotype sharing (Fig. 8).

Each recurrent V(D)J framework contained a diverse collection of CDR3 amino-acid sequences, indicating that recurrent gene-segment usage was not attributable to a single shared clonotype across animals (Fig. 8A, B). Nevertheless, all six V(D)J frameworks contained public CDR3 sequences detected in more than one mouse. The three selected IGH frameworks contained 91, 68, and 39 public CDR3 amino-acid sequences, respectively, whereas the corresponding TRB frameworks contained 130, 87, and 67 public sequences. Public CDR3s represented 25.5-36.5% of unique CDR3 amino-acid sequences within the selected IGH frameworks and 37.7-44.4% within the selected TRB frameworks (Fig. 8C, D). Thus, within these selected recurrent V(D)J frameworks, repertoire sharing extended beyond gene-segment usage to identical CDR3 amino-acid sequences in subsets of animals.

We next examined the extent of cross-animal sharing of the most broadly distributed public CDR3 sequence within each selected V(D)J framework. For IGH, these sequences were detected in 12, 61, and 18 mice for V1, V2, and V3, respectively (Fig. 8E). The corresponding TRB sequences were detected in 37, 26, and 21 mice, respectively (Fig. 8F). These findings identify individual public CDR3 amino-acid sequences that are broadly shared across animals, although the extent of sharing differed substantially among the selected V(D)J frameworks.

Finally, we examined the distribution of these broadly shared public CDR3 sequences across naïve spleen, stroke spleen, and chronic infarct repertoires (Fig. 8G, H). The selected IGH public sequences exhibited heterogeneous compartmental distributions: some were detected more frequently in infarct repertoires, whereas others were also broadly represented in peripheral spleen repertoires. The selected public TRB sequences were similarly detected across tissue compartments, with their distributions differing among V(D)J frameworks. Thus, public CDR3 sharing was not uniformly restricted to the chronic infarct.

Together, these findings indicate that recurrent IGH and TRB V(D)J frameworks contain substantial CDR3 sequence diversity together with subsets of public CDR3 amino-acid sequences shared across animals. The heterogeneous tissue distribution of the most broadly shared sequences further indicates that public clonotypes are not uniformly associated with the chronic infarct. Rather, chronic infarct repertoires comprise diverse private sequences together with public clonotypes that differ in the extent of cross-animal sharing and compartmental distribution. Thus, recurrence at the level of V(D)J gene usage and CDR3 sequence sharing does not necessarily indicate selective association with the chronic infarct.

## 4. Discussion

Persistent adaptive immune responses are increasingly recognized as a feature of chronic ischemic stroke, yet their repertoire-level organization remains incompletely understood. Extending our previous TCR analyses [18], the present study demonstrates that repertoire-level organization after stroke also encompasses the B-cell compartment. Chronic infarcts exhibited increased IGH clonal concentration relative to splenic repertoires, recurrent V(D)J gene usage among dominant clonotypes, public CDR3 sequences within selected receptor frameworks, and a positive association between B- and T-cell repertoire concentration within matched lesions. However, the magnitude of IGH remodeling varied substantially among animals and across age- and sex-defined cohorts, and individual infarcts displayed distinct paired IGH and TRB architectures. These findings indicate that persistent adaptive immunity after stroke is organized rather than stochastic, but that the form and extent of this organization are biologically heterogeneous.

Evidence that adaptive humoral immune responses can develop after stroke dates back several decades, when oligoclonal IgG bands were reported in the cerebrospinal fluid of selected patients following cerebral infarction [36]. Although intrathecal oligoclonal bands and tissue BCR repertoires represent distinct measures of humoral immunity, both are consistent with non-random adaptive immune responses.

Our findings extend these observations by showing that B-cell accumulation within the chronic infarct can be accompanied by selective concentration of the local IGH repertoire. The histological increase in lesion-associated B cells establishes their accumulation within chronic infarcts, whereas repertoire sequencing demonstrates that the composition of this accumulated population can range from broadly distributed to clonally concentrated across individual animals adding a repertoire-level dimension to reports of ectopic lymphoid aggregates and tertiary lymphoid-like structures containing B and T cells in multiple sclerosis [37], experimental spinal cord injury [38], and chronic experimental stroke [23]. However, the present data do not establish that dominant B-cell clones arise within these structures, nor do they determine whether repertoire remodeling is protective, pathogenic, or functionally neutral. Resolving how clonal architecture relates to lymphoid organization, antibody production, persistent neuroinflammation, and long-term recovery will require spatially resolved analyses that link receptor sequence to cellular phenotype, anatomical location, antigen specificity, and function.

The magnitude and consistency of IGH repertoire remodeling varied across age and sex. Within chronic infarcts, young females generally exhibited lower clonality than the other age and sex-defined groups, although occasional animals showed marked clonal concentration. Young and aged males exhibited more frequent infarct-associated increases relative to their corresponding splenic compartments. In aged females, however, relatively elevated clonality was also evident in naïve and stroke spleens, indicating that repertoire concentration in this group was not associated with the infarct. These observations suggest that age- and sex-associated differences in baseline peripheral repertoire composition may influence the apparent tissue specificity of chronic post-stroke B-cell responses. These findings should be interpreted cautiously because the study was not powered to assess age-by-sex interactions, and variation in productive sequence recovery may have affected estimates in some groups.

This heterogeneity has important implications for experimental design. In a small cohort, one or two animals with unusually high or low clonal expansion could disproportionately influence the group result. Similarly, an experiment restricted to young males could identify a relatively consistent pattern that is weaker or more variable in females, aged animals, or other biological contexts. Conversely, a negative result in a small cohort may reflect insufficient power in the setting of substantial biological variation rather than the absence of repertoire remodeling. Findings from low-powered studies or experiments using a single age or sex should therefore be generalized cautiously. Larger factorial studies with adequate sample sizes, formal interaction analyses, and careful control of repertoire sampling depth will be required to determine whether the observed patterns reflect differences in B-cell recruitment, local clonal expansion, baseline repertoire composition, or technical sampling.

Further analysis of immunoglobulin light-chain loci provided complementary, locus-dependent evidence of repertoire remodeling. IGK largely resembled IGH, with variable clone-fraction profiles and tissue-associated increases in clonality that were most consistent in male cohorts. In contrast, IGL showed a more concentrated clone-fraction distribution, with the ten most abundant clonotypes accounting for a larger fraction of the repertoire across animals. However, significant tissue effects in IGL clonality were limited to selected groups. Together, these data indicate that heavy- and light-chain loci share some post-stroke repertoire features but do not remodel identically. Because sequencing was performed on bulk genomic DNA, similarities between IGH and IGK reflect population-level patterns and do not imply native heavy–light chain pairing within individual B cells.

Building on our previous identification of infarct-associated T-cell oligoclonality [18], paired analysis demonstrated that IGH and TRB clonality were positively associated across matched chronic infarcts. Lesions with greater B-cell repertoire concentration therefore tended also to exhibit greater T-cell repertoire concentration. This relationship could reflect shared lesion-level factors, including antigen availability, inflammatory burden, tissue damage, chemokine expression, vascular permeability, or the development of local lymphoid niches. However, the correlation does not establish direct B-cell and T-cell interaction, shared antigen specificity, directionality, or causal dependence. Individual infarcts also displayed discordant paired architectures, including lesions with concentrated T-cell repertoires but comparatively distributed B-cell repertoires. Chronic infarcts therefore differ not only in the overall magnitude of adaptive immune remodeling but also in the relative concentration of B- and T-cell clonal expansion. The whole-cohort IGH and TRB repertoire maps further illustrate this spectrum of lesion-level organization across individual animals.

V(D)J analysis identified an additional level of repertoire organization beyond overall clonal concentration. Selected IGH V(D)J frameworks recurred among dominant infarct clonotypes within each age- and sex-defined cohort, and some frameworks were represented across all four biological groups. TRB repertoires showed a similar pattern, indicating that repeated receptor-gene usage is evident in both B- and T-cell compartments despite substantial overall sequence diversity. Recurrent frameworks occurred at different positions within the dominant clone hierarchy rather than being uniformly represented by the most expanded clonotype. Thus, the shared feature across animals was the repeated use of selected receptor-gene combinations within otherwise heterogeneous patterns of clonal expansion, rather than expansion of a single stereotyped dominant clone.

These findings must nevertheless be interpreted conservatively. The same V(D)J combination can generate many CDR3 sequences with distinct antigen specificities, and intrinsic biases in recombination can cause some V, D, and J combinations to occur more frequently than others [39]. Recurrent frameworks therefore identify reproducible receptor-gene patterns but do not independently demonstrate recognition of a shared antigen or antigen-driven selection. Formal comparison with background V(D)J usage frequencies, generation probabilities, and appropriately matched peripheral repertoires will be needed to determine whether particular frameworks are selectively enriched within chronic infarcts.

CDR3 amino-acid sequence analysis further examined whether recurrent receptor-gene usage extended to sequence-level sharing across animals. Selected recurrent IGH and TRB frameworks contained extensive CDR3 diversity, indicating that repeated framework usage was not attributable to expansion of a single shared clonotype across the cohort. Nevertheless, each selected framework contained public CDR3 amino-acid sequences detected in multiple animals, including some shared across substantial numbers of mice. The most broadly shared public clonotypes also showed heterogeneous distributions across naïve spleen, stroke spleen, and chronic infarct repertoires. Some were detected more frequently in infarcts, whereas others were broadly represented in both peripheral and lesion compartments.

Public receptor sequences can arise through convergent recombination, relatively high probabilities of generation, or shared immune exposures [39, 40]. Identical CDR3 amino-acid sequences therefore do not establish common antigen recognition or specificity for brain-derived antigens. Interestingly, the most broadly shared IGH public clonotypes were each encoded by a single nucleotide sequence despite being detected in multiple animals. Together with their occurrence in naïve repertoires, this observation raises the possibility that some recurrent antibody clonotypes represent pre-existing germline-associated or natural antibody specificities that are recruited or expanded after stroke [41]. This interpretation remains speculative and will require direct functional validation through recombinant antibody generation, antigen-binding studies, and assessment of somatic hypermutation and isotype usage. Overall, the coexistence of extensive sequence diversity, public clonotypes, and heterogeneous compartmental distributions indicates that chronic post-stroke repertoires contain both shared and individual-specific components rather than a single stereotyped adaptive immune response.

Several additional limitations should be considered. Bulk repertoire sequencing does not identify the phenotype, activation state, anatomical location, or functional activity of individual clones. It also cannot distinguish local proliferation from selective recruitment or prolonged retention of clonotypes expanded elsewhere. Differences in tissue cellularity and template recovery may affect repertoire estimates, particularly in groups with lower sequence recovery. The study was conducted at a single chronic endpoint, limiting conclusions about when clonal expansion begins, how stable it remains, and whether B- and T-cell repertoire organization develops synchronously. Finally, recurrence and public sequence sharing do not identify the antigens driving the response. Longitudinal, spatial, and single-cell approaches that link paired receptor sequences to transcriptional phenotype, immunoglobulin isotype, anatomical niche, and antigen reactivity will be necessary to define the origins and consequences of chronic post-stroke adaptive immunity.

These findings broaden how persistent adaptive immunity after stroke can be investigated. Chronic post-stroke neuroinflammation has traditionally been characterized through immune-cell abundance, localization, and phenotype. Adaptive immune receptor repertoires provide a complementary molecular record of clonal selection and expansion that cannot be inferred from histology or transcriptional state alone. The publicly deposited, sample-resolved repertoire dataset and accompanying analytical workflows also provide a resource for testing alternative measures of repertoire organization, comparing future post-stroke cohorts, and investigating candidate antigenic drivers. By integrating B- and T-cell repertoire analyses across biologically and experimentally diverse cohorts, the present study demonstrates that the chronic infarct is not simply a site of persistent lymphocyte infiltration, but a neuroimmune environment containing localized and reproducibly detectable, yet highly heterogeneous, adaptive immune repertoire remodeling.

In conclusion, persistent adaptive immunity after stroke is organized at multiple repertoire levels, including clonal concentration, recurrent receptor-gene usage, public CDR3 sequence sharing, and paired B- and T-cell repertoire organization. This organization is reproducible across experimental contexts but varies substantially among individual lesions and across age- and sex-defined cohorts. Because B-cell repertoire remodeling is biologically heterogeneous, small, single-sex, or underpowered studies may miss true differences or exaggerate patterns driven by only a few animals. As a result, findings from such studies should be interpreted cautiously and validated in larger, balanced cohorts that can directly test age and sex effects. Although the present study does not establish shared antigen recognition or define the functional consequences of clonal expansion, it provides a framework for determining how persistent adaptive immune cells accumulate and organize in the chronic infarct may influence chronic neuroinflammation and long-term recovery after stroke.

## Supplementary Material

1

## Data Availability

The datasets generated and analyzed during the current study have been deposited in the Zenodo repository (DOI: 10.5281/zenodo.21799549) and is publicly available. The repository contains the filtered raw immunoglobulin heavy-chain (IGH), immunoglobulin kappa light-chain (IGK), and immunoglobulin lambda light-chain (IGL) repertoire datasets from the age- and sex-stratified single-site cohort, together with the resolved IGH V(D)J repertoire data from the model-stratified multisite cohort. All source data underlying the figures, where applicable, are provided as Supplementary tables within this manuscript.
