# Supplementary material for: Integrated B and T Cell Repertoire Analysis Reveals Organized but Heterogeneous Adaptive Immunity in Chronic Experimental Stroke
Source: Res Sq. 2026 Aug 27:rs.3.rs-10695363. Preprint. [Version 1] doi: 10.21203/rs.3.rs-10695363/v1 (PMC13532746; doi:10.21203/rs.3.rs-10695363/v1)
Supplement: Supplementary file 1 [file NIHPPrs10695363v1-supplement-1.pdf]

Supplementary Figures

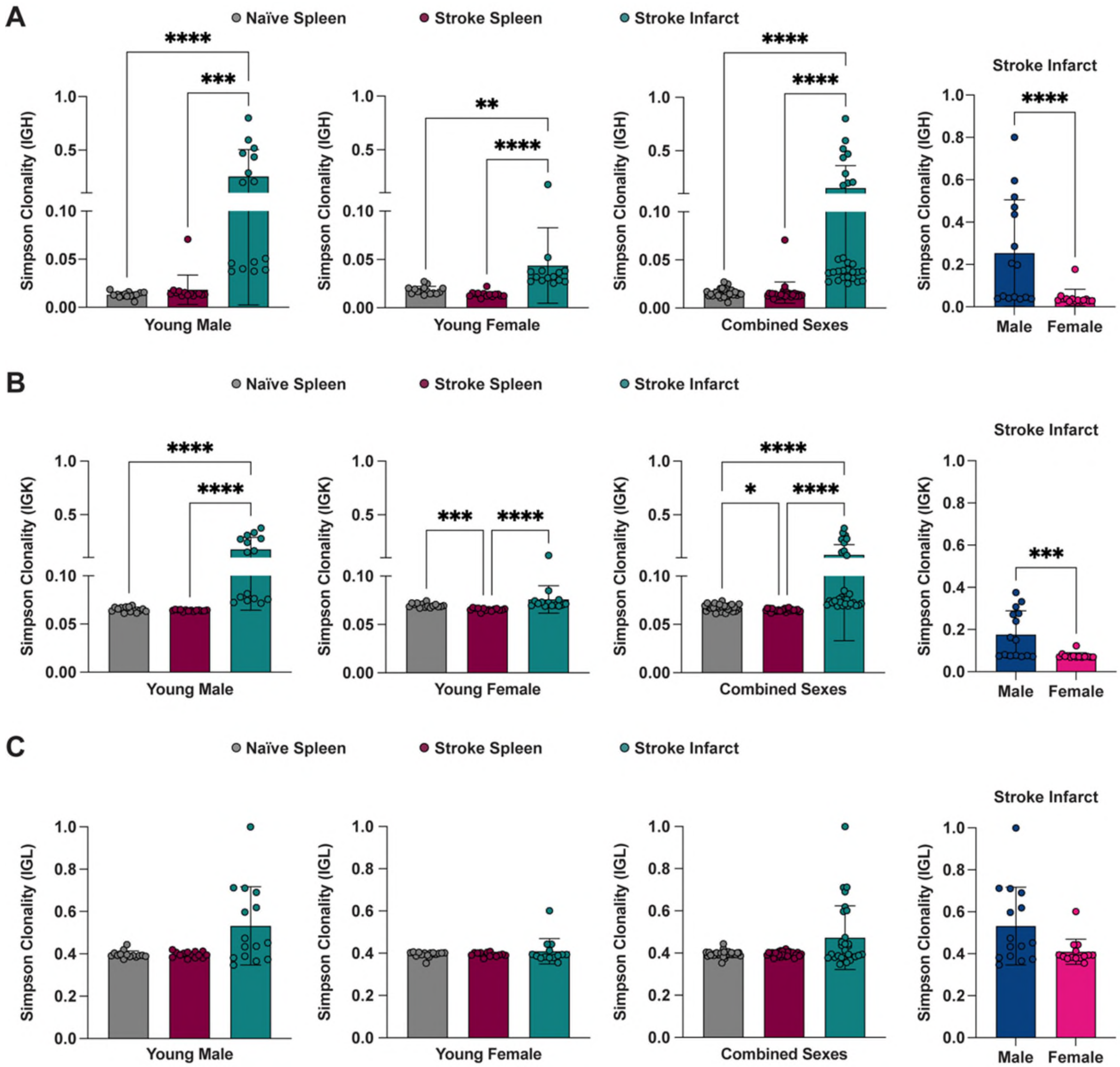

Supplementary Figure 1. Tissue- and sex-dependent differences in BCR clonality in young mice. Simpson clonality of (A) IGH, (B) IGK, and (C) IGL repertoires was compared across naïve spleen, spleen from stroked mice, and chronic infarct tissue in young mice. In young males, infarct repertoires showed significantly higher IGH and IGK clonality than both naïve and post-stroke spleen. In young females, infarct IGH clonality was significantly higher than that of both splenic compartments. For IGK, stroke spleens

showed lower clonality than naïve spleens, whereas infarct clonality was higher than stroke-spleen clonality. When sexes were combined, infarct IGH clonality was significantly higher than that of both splenic compartments. Combined IGK analysis identified differences among all three tissue groups, including lower clonality in stroke spleen than naïve spleen and higher clonality in infarct than either splenic compartment. No significant tissue differences were detected for IGL. Direct comparison of chronic infarct repertoires showed significantly higher IGH and IGK clonality in young males than in young females, whereas IGL clonality did not differ significantly by sex. Each point represents an individual mouse. Data are presented as mean ± SD. *$P < 0.05$; **$P < 0.01$; ***$P < 0.001$; ****$P < 0.0001$.

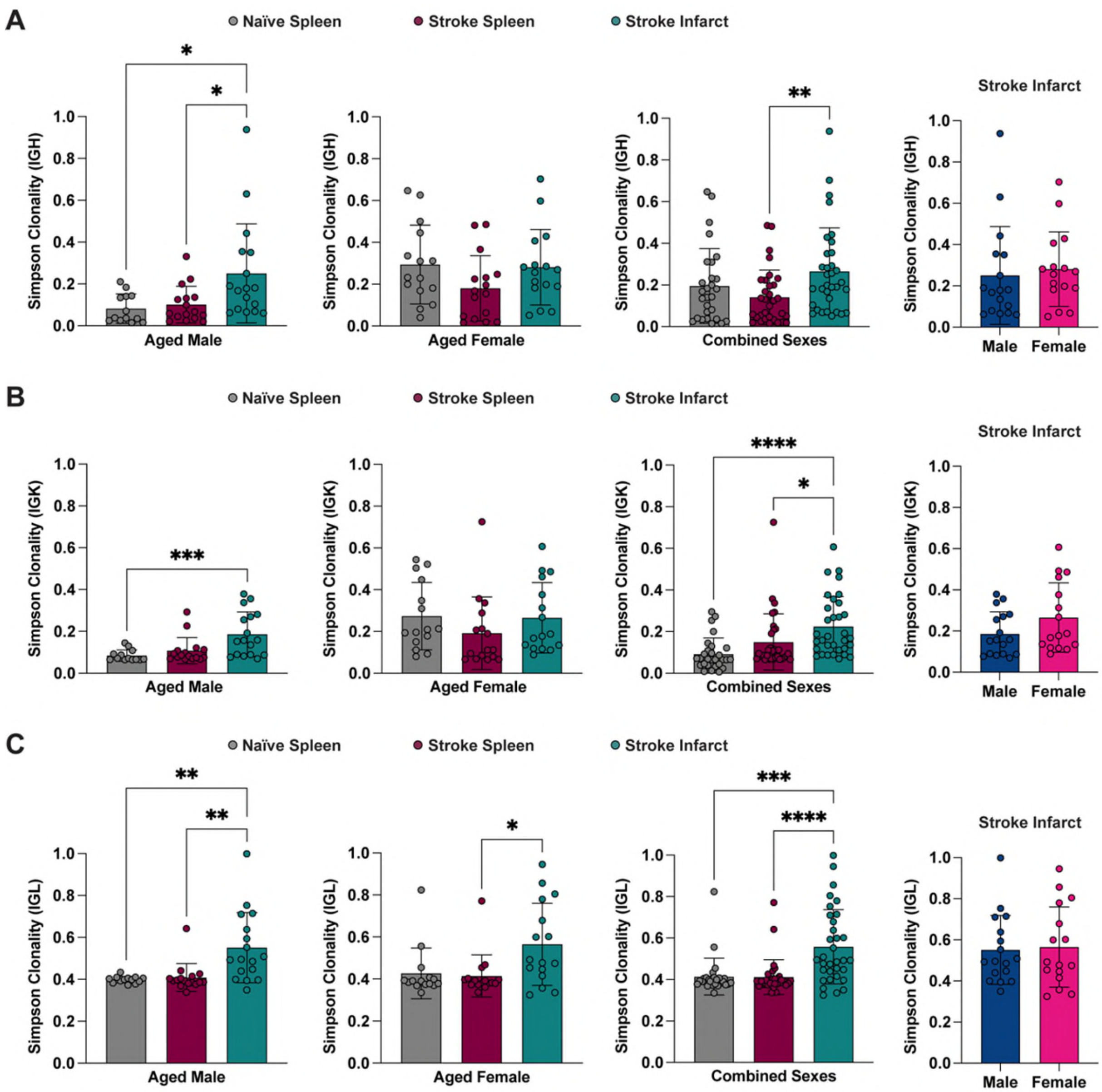

Supplementary Figure 2. Tissue- and sex-dependent differences in BCR clonality in aged mice. Simpson clonality of (A) immunoglobulin heavy-chain (IGH), (B) immunoglobulin kappa light-chain (IGK), and (C) immunoglobulin lambda light-chain (IGL) repertoires was compared among naïve spleen, spleen from stroked mice, and chronic infarct tissue in aged mice. In aged males, infarct repertoires exhibited significantly higher IGH and IGL clonality than both naïve and stroke spleens, whereas IGK clonality was significantly higher in infarct tissue than in naïve spleen only. In aged

females, IGH and IGK clonality did not differ significantly among tissue compartments, whereas IGL clonality was significantly higher in infarct tissue than in stroke spleen. When both sexes were combined, infarct repertoires exhibited significantly higher IGK and IGL clonality than both naïve and stroke spleens, whereas combined IGH clonality was significantly higher in infarct tissue than in stroke spleen only. Direct comparison of chronic infarct repertoires identified no significant sex differences in IGH, IGK, or IGL clonality. Each point represents an individual mouse. Data are presented as mean ± SD. *$P < 0.05$; **$P < 0.01$; ***$P < 0.001$; ****$P < 0.0001$.

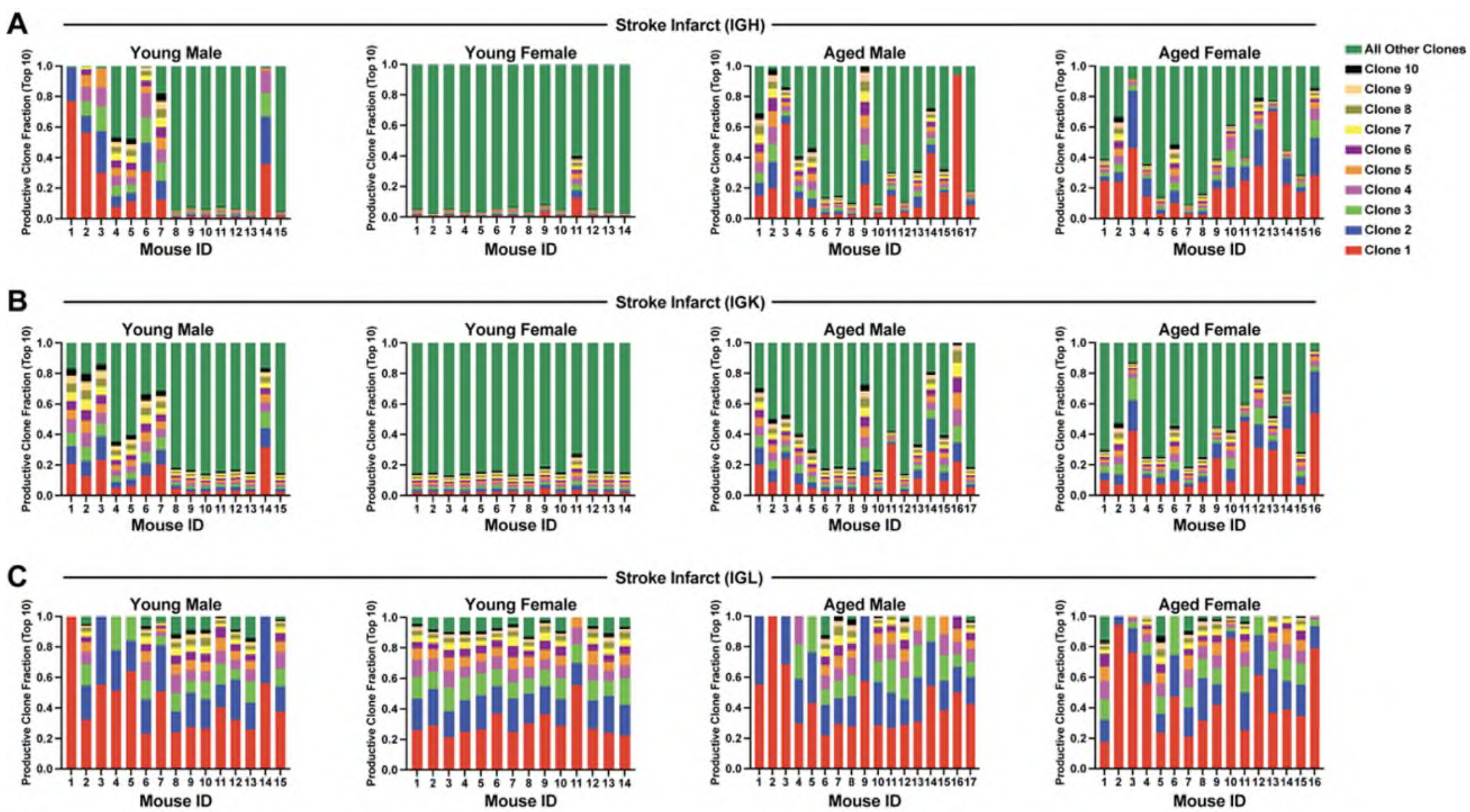

Supplementary Figure 3. Top-clone distribution reveals immunoglobulin locus-specific patterns of clonal architecture. Stacked bar graphs show the relative abundance of the 10 most abundant productive clonotypes and the combined abundance of all remaining clonotypes within each infarct repertoire. Each bar represents an individual mouse and sums to 1.0. Clone 1 through Clone 10 represent clonotypes ranked by decreasing abundance within each sample, while the green segment represents the combined contribution of all clonotypes outside the top 10. **(A)** IGH, **(B)** IGK, and **(C)** IGL repertoires demonstrate substantial inter-animal variation in clonal concentration across age and sex groups, with visually distinct patterns of top-clonotype contribution among immunoglobulin loci.

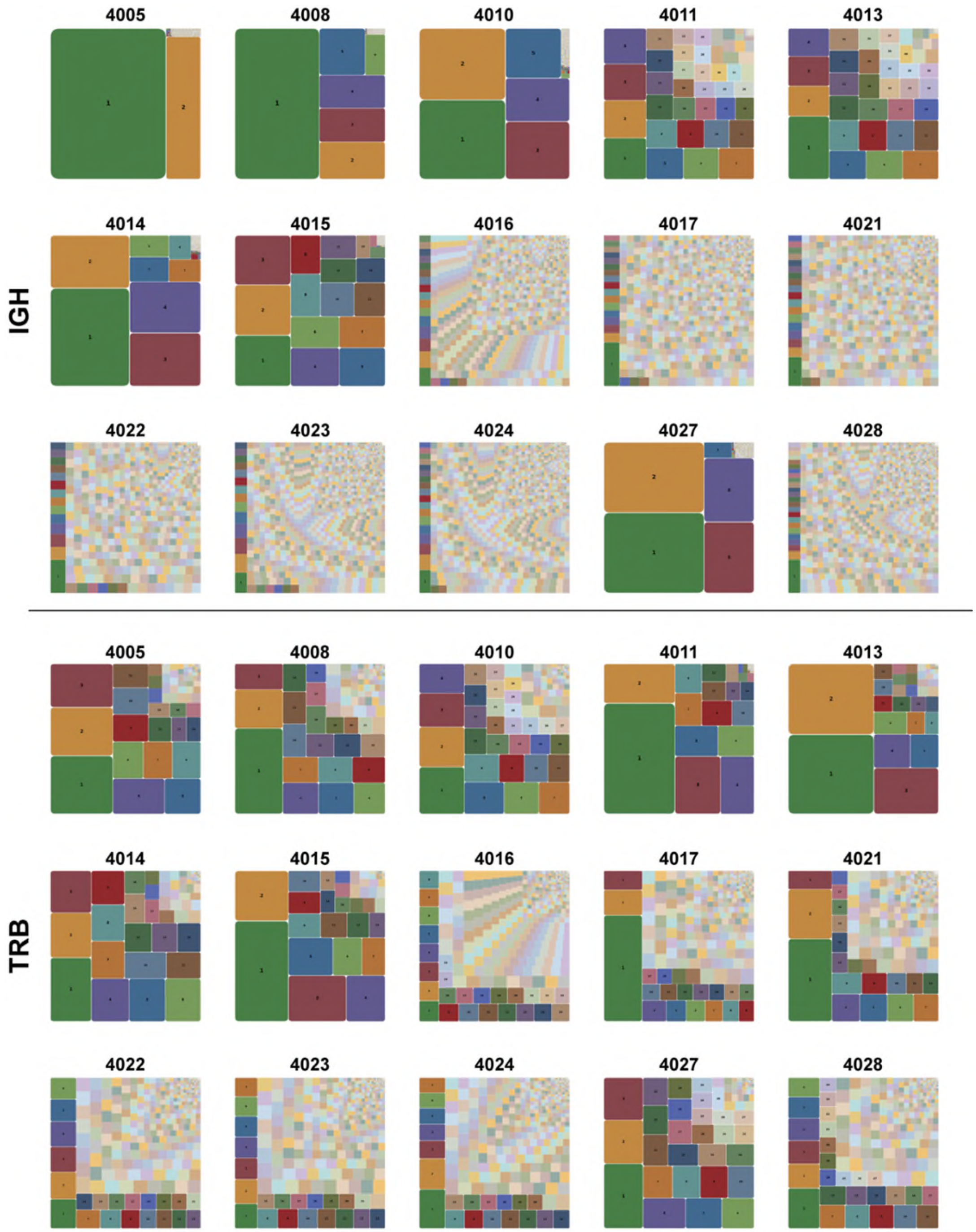

1188

1189 **Supplementary Figure 4. Paired treemaps of young male IGH-TRB repertoires.** The
1190 numbers above each treemap represent individual mouse IDs.

1191

1192

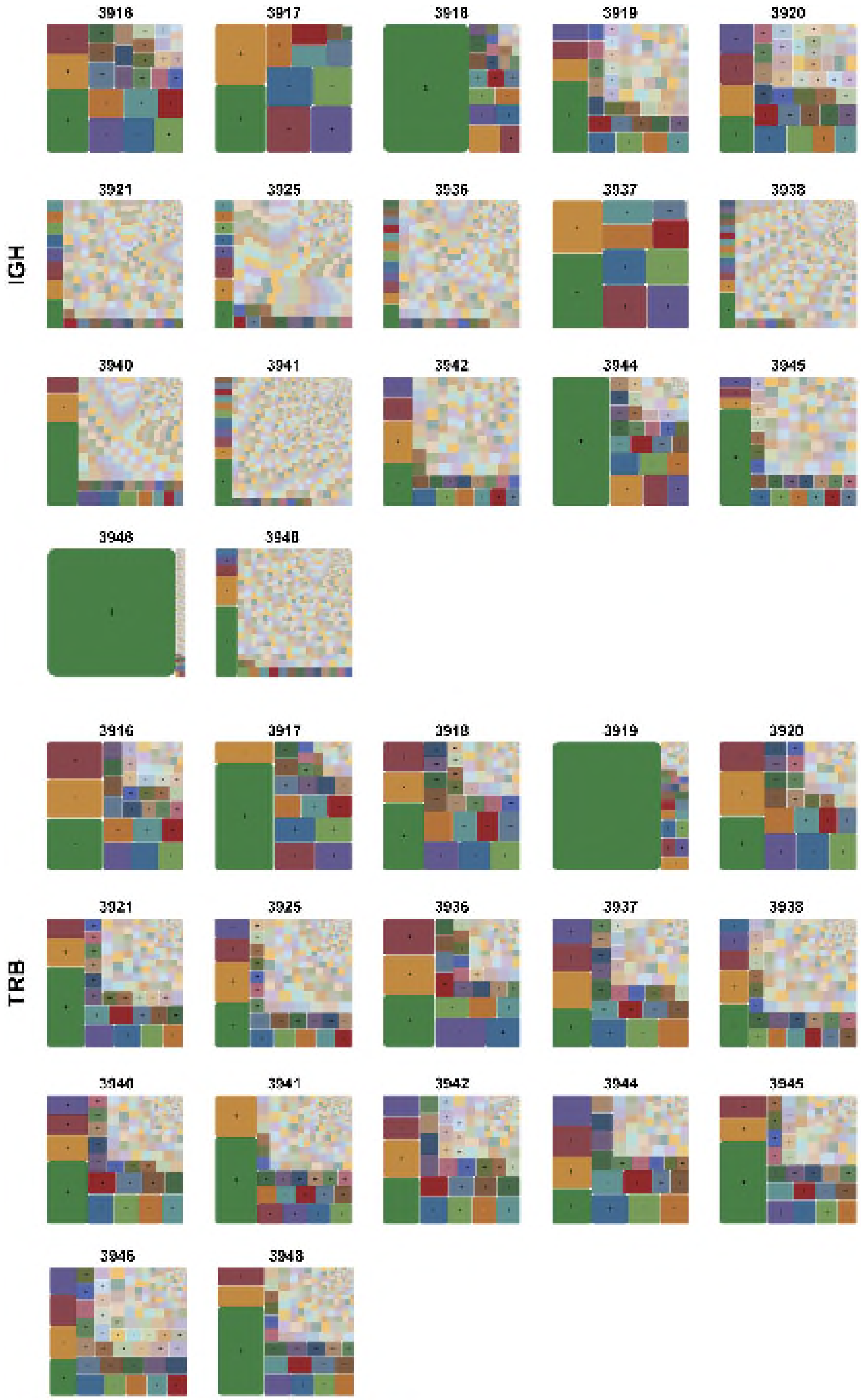

Supplementary Figure 5. Paired treemaps of aged male IGH-TRB repertoires. The numbers above each treemap represent individual mouse IDs.

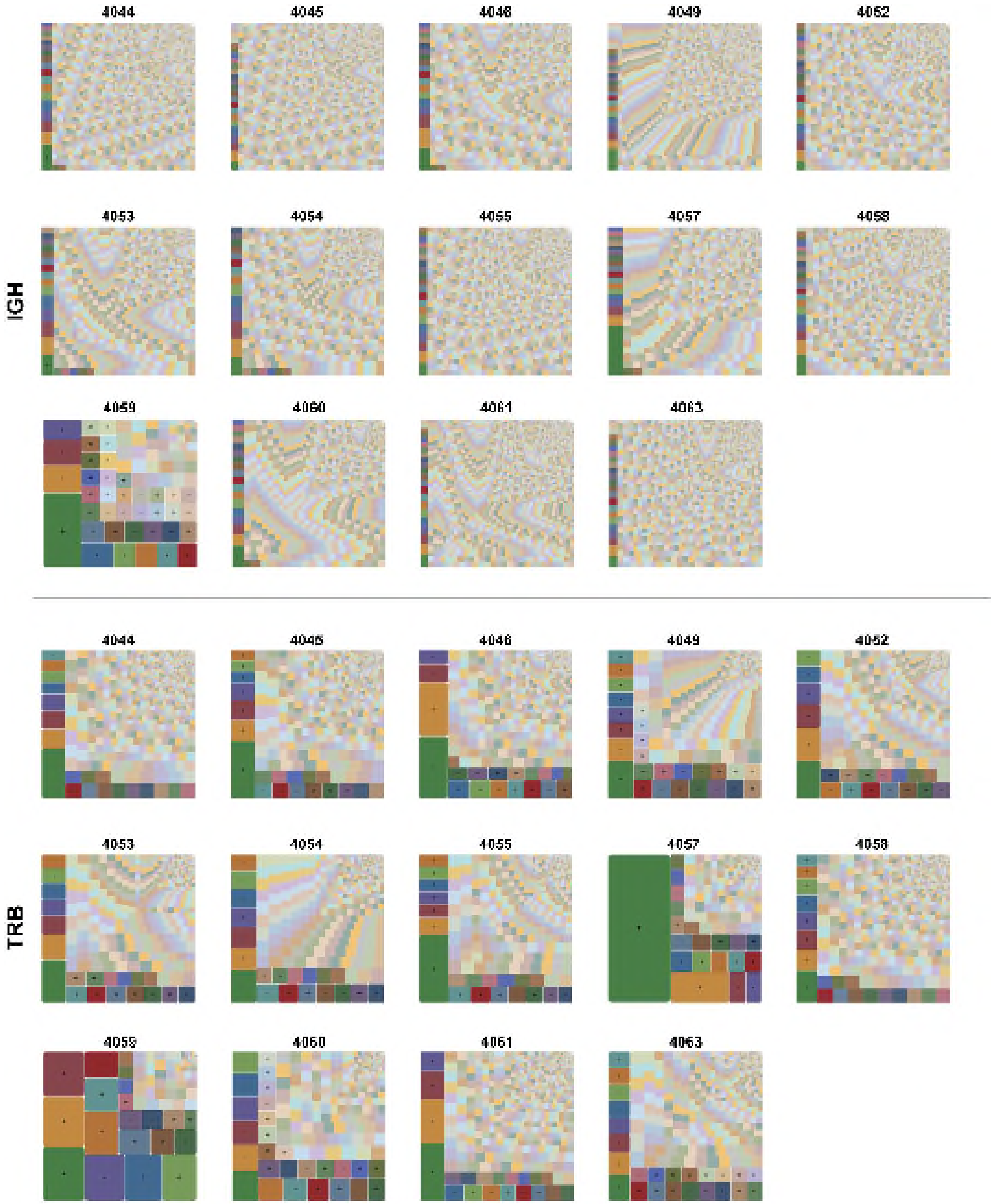

Supplementary Figure 6. Paired treemaps of young female IGH-TRB repertoires.

The numbers above each treemap represent individual mouse IDs.

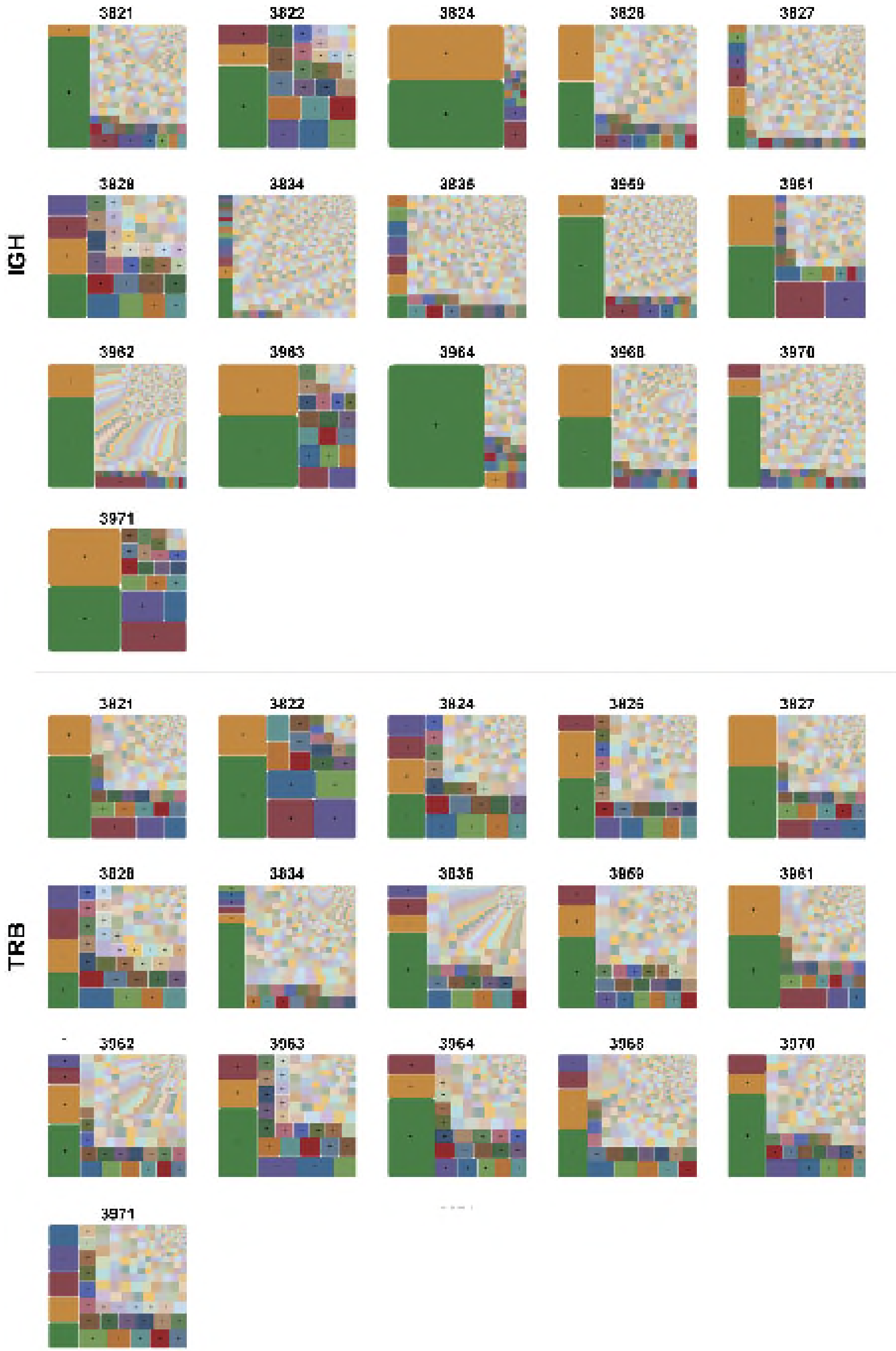

Supplementary Figure 7. Paired treemaps of aged female IGH-TRB repertoires. The numbers above each treemap represent individual mouse IDs.
